# Native Ambient Mass Spectrometry Enables Analysis of Intact Endogenous Protein Assemblies up to 145 kDa Directly from Tissue

**DOI:** 10.1021/acs.analchem.1c05353

**Published:** 2022-03-31

**Authors:** Oliver J. Hale, James W. Hughes, Emma K. Sisley, Helen J. Cooper

**Affiliations:** School of Biosciences, University of Birmingham, Edgbaston, Birmingham B15 2TT, U.K.

## Abstract

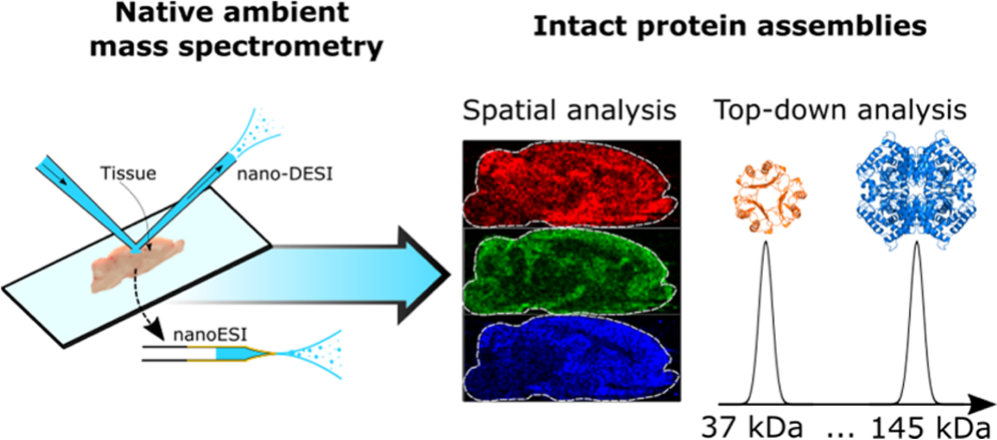

Untargeted label-free interrogation of proteins in their functional form directly from their physiological environment promises to transform life sciences research by providing unprecedented insight into their transient interactions with other biomolecules and xenobiotics. Native ambient mass spectrometry (NAMS) shows great potential for the structural analysis of endogenous protein assemblies directly from tissues; however, to date, this has been limited to assemblies of low molecular weight (<20 kDa) or very high abundance (hemoglobin tetramer in blood vessels, RidA homotrimer in kidney cortex tissues). The present work constitutes a step change for NAMS of protein assemblies: we demonstrate the detection and identification of a range of intact endogenous protein assemblies with various stoichiometries (dimer, trimer, and tetramer) from a range of tissue types (brain, kidney, liver) by the use of multiple NAMS techniques. Crucially, we demonstrate a greater than twofold increase in accessible molecular weight (up to 145 kDa). In addition, spatial distributions of protein assemblies up to 94 kDa were mapped in brain and kidney by nanospray desorption electrospray ionization (nano-DESI) mass spectrometry imaging.

## Introduction

Native ambient mass spectrometry (NAMS) integrates native mass spectrometry,^1−4^ in which noncovalent interactions present in solution phase are retained in the gas phase, with ambient mass spectrometry, in which biological substrates are analyzed directly without (or with very little) sample pretreatment. The benefits of NAMS for proteins are that both structural and spatial information can be obtained simultaneously. Liquid extraction surface analysis (LESA),^5−10^ desorption electrospray ionization (DESI),^11,12^ and more recently nanospray desorption electrospray ionization (nano-DESI)^13,14^ have all been demonstrated for intact protein analysis under native-like conditions. Two techniques, LESA and nano-DESI, have so far been applied to NAMS of proteins in tissue.^6,7,13,14^ The former is the more flexible technique as the sampling and ionization steps can be decoupled, and each step can be optimized independently. Both LESA and nano-DESI can be applied for spatial mapping of proteins, i.e., mass spectrometry imaging (MSI), but nano-DESI is generally preferred as it offers higher spatial resolution.

For small molecules and lipids, recent technological and methodological developments have transformed MSI into a powerful spatial analysis technique.^15−17^ In contrast, MSI of proteins is rarely performed and is challenging due to reasons including their greater mass (and mass range). Ionization techniques commonly used for MSI, matrix-assisted laser desorption/ionization (MALDI),^18,19^ DESI,^20−23^ nano-DESI,^24−27^ LESA,^28,29^ and liquid microjunction surface sampling (LMJ-SS)^22,30,31^ have all been applied to intact protein analysis under denaturing conditions but are limited to proteins with molecular weight (MW) < 30 kDa. Furthermore, any tertiary or quaternary structure is lost under denaturing conditions. Both of these limitations can be addressed by NAMS, which enables structural analysis of proteins directly from tissues. For example, we have previously shown how protein collision cross sections determined by NAMS are in good agreement with those determined by X-ray crystallography;^5,13^ however, to date, just three protein assemblies have been detected and identified from tissue by NAMS. These include the highly abundant hemoglobin tetramer (∼64 kDa) and RidA trimer (∼43 kDa) and the low-molecular-weight S100-A6 dimer (∼19 kDa). Oligomeric protein assemblies are highly prevalent in nature, with around half of proteins with experimentally determined structures known to form homomeric assemblies.^32^

Here, we present a comprehensive analysis of endogenous protein assemblies detected and identified in brain, kidney, and liver tissues. These assemblies span a broad range of molecular weights (up to 145 kDa) and exhibit various stoichiometries (dimer, trimer, tetramer). Analysis of larger protein assemblies was achieved through tuning ion optics and gas pressures in the mass spectrometers for improved high *m*/*z* transmission and a focus on improved nano-DESI and nanoelectrospray performance. The results illustrate the broad utility of the emerging field of native ambient mass spectrometry.

## Experimental Section

### Materials

MS-grade water was purchased from Fisher Scientific (Loughborough, U.K.). HPLC-grade ammonium acetate was purchased from J.T. Baker (Deventer, Netherlands). The detergents C_8_E_4_ and LDAO were purchased from Sigma-Aldrich (Gillingham, U.K.). Calibration solutions were obtained from Thermo Fisher Scientific (San Jose, CA). Solvent systems consisted of 200 mM aqueous ammonium acetate, with concentrations of detergent between 0.5× and 2× their critical micelle concentrations (specified with relevant results). No organic solvents were included. The solvent system used for producing ion images was ammonium acetate (200 mM) + 0.5× CMC C_8_E_4_ detergent. Nitrogen (>99.995%) and helium (>99.996%) gases used on the mass spectrometer were obtained from BOC (Guildford, U.K.). Harris hematoxylin, acid alcohol, industrial denatured alcohol, Scott’s tap water substitute, xylene, and eosin (1% aqueous) were purchased from pfm Medical (Cheshire, U.K.). DPX was purchased from CellPath (Powys, U.K.).

### Animal Tissues

Kidney, liver, and brain tissues from vehicle-dosed (0.5% HPMC and 0.1% Tween 80 in water), i.e., control, adult male Han Wistar rats were a kind gift of Prof Richard Goodwin (Astra Zeneca). The animal was euthanized 2 h post dose. Dissection was performed by trained Astra Zeneca staff (project license PP77366793, procedure number 3). Kidneys were snap-frozen in isopropanol over dry ice. Other organs were snap-frozen in isopentane over dry ice. All tissues were stored at −80 °C and sectioned at −22 °C to a thickness of 10 μm with a CM1810 Cryostat (Leica Microsystems, Wetzlar, Germany) and thaw-mounted to glass microscope slides. Sections were stored at −80 °C until use. Tissue sections were not washed prior to analysis to avoid delocalization and structural disruption of proteins. Serial sections of the brain and kidney were subjected to hematoxylin and eosin staining, as detailed in Table S1, Supporting Information.

### Nano-DESI

The nano-DESI ion source (described in the Supporting Information) was coupled to an Orbitrap Eclipse mass spectrometer (Thermo Scientific, San Jose, CA).

#### Nano-DESI MSI

The solvent flow rate was 2 μL/min, and the electrospray voltage was 1.3 kV. The nano-DESI probe was moved laterally at 20 μm/s for rat brain analysis and 10 μm/s for rat kidney analysis with a line scan spacing of 200 μm.

#### Nano-DESI MS*^n^*

For MS*^n^* (i.e., proton transfer charge reduction (PTCR) and higher-energy collisional dissociation (HCD)), settings were the same as above but with a probe movement rate of 1–2 μm/s.

### LESA Microextraction and Nanoelectrospray Ionization

In these experiments, LESA microextraction was followed by sample collection in a well plate. The sample was then loaded into a gold-coated borosilicate nanoelectrospray emitter, i.e., the LESA sampling and ionization processes were decoupled. These experiments are referred to as “nanoESI” throughout this Article. Details of the microextraction are given in the Supporting Information.

Borosilicate glass capillaries were prepared in house using a P-1000 pipette puller (Sutter Instrument) before coating with gold using a sputter coater (Agar Scientific Ltd.).

Sample-loaded tips were inserted into a nanospray ion source equipped with the static spray option (Thermo) attached to either of the mass spectrometers described below. The electrospray voltage for the tips was typically in the range of 1.0–1.2 kV and performed with no additional backing pressure. The use of borosilicate emitters improved nanoelectrospray stability, duration, and signal intensity when compared with that of chip-based nanoESI. This observation can be attributed to the narrower spray orifice (1–2 μm) and the tapered geometry of the borosilicate emitter versus the square-cut geometry of the chip-based nanoESI emitters.^33^

### Mass Spectrometry

Mass spectrometry was performed on an Orbitrap Eclipse (Thermo Scientific) equipped with the HMR*^n^* option and a Q-Exactive HF (“QE-HF”, Thermo Scientific). The Q-Exactive HF featured customized, research-only instrument control software, which gave access to extended trapping gas, mass resolution, *m*/*z* range, and quadrupole isolation parameters, supplied by Thermo Fisher Scientific. Trapping gas was set to 5 (arbitrary units). The voltages of the source ion optics were optimized to enhance the transmission of higher *m*/*z* ions with an inject flatapole offset, an interflatapole lens, and a bent flatapole set to 7, 6, and 5 V, respectively. The mass spectrometers were calibrated with Flexmix (Eclipse) or Calmix (QE-HF) (both from Thermo Fisher). Ion transfer tube temperatures were 275 °C (Eclipse) and 250 °C (QE-HF).

Two ion source optics settings proved critical in enabling higher mass analysis on the Orbitrap Eclipse: source dissociation voltage (SDV) and source compensation value (SCV). (Note, the former is referred to as “source-induced dissociation, sid” in the vendor terminology, but we have refrained from using this nomenclature due to the potential confusion with the established tandem mass spectrometry (MS/MS) technique “surface-induced dissociation, SID”.) The benefits of optimizing these settings for protein analysis have been detailed by others.^1,34^ Additionally, the ion routing multipole (IRM) chamber was set to a pressure of 20 mTorr. This pressure is higher than the “standard” operating pressure (8 mTorr) and acts to improve the trapping of high mass ions and preserve protein assemblies.

For the rat brain MSI, source ion optics were set to SDV = 130 V and SCV = 7% based on the observation of multiple abundant signals between approx. *m*/*z* 3920 and 5000. For rat kidney MSI, SDV = 80 V and SCV = 11% based on the observation of multiple abundant signals between approx. *m*/*z* 3900 and 5400.

PTCR MS^2^ was performed using the Orbitrap Eclipse and used for reducing protein ion charge states by the gas-phase reaction with perfluoroperhydrophenanthrene anions in the high-pressure cell of the linear ion trap. Target protein ions were isolated by *m*/*z* in the ion trap prior to PTCR. Reagent anion automatic gain control (AGC) target was 2 × 10^5^ charges, with reaction time varied according to protein ion *m*/*z* and typically in the range of 0.5–5 ms. Injection time was 200–1500 ms.

On both instruments, the orbitrap mass analyzers were used in low-resolution (7500 at *m*/*z* 200; transient length of 16 ms) modes for full scan and PTCR MS^2^ intact protein analysis. Injection times were typically 500–1500 ms, with an AGC target of 5 × 10^6^ charges. For top-down fragmentation by collisional activation (HCD and CID), higher-resolution (up to 500,000 at *m*/*z* 200) data were acquired to resolve the isotopic distribution of fragment ions. Isolation window widths for HCD, CID, and PTCR were up to *m*/*z* 30, typically set wider at higher *m*/*z* to improve ion transmission. In some cases where sequence ions were difficult to obtain from the intact complex, protein complexes were dissociated into subunits in the ion source region of the mass spectrometer, followed by the isolation of subunit ions in the ion trap for subsequent collisional dissociation (“pseudo-MS^3^”). True MS^3^ was performed on the Orbitrap Eclipse using the ion trap and IRM for multistage isolation and fragmentation. Injection time for MS*^n^* experiments was typically 500–1500 ms, with an AGC target of up to 1 × 10^6^ charges.

### Ion Image Generation

Ion images were produced by conversion of Thermo raw files for each line scan to a single imzML file by Firefly (v.3.2.0.23, Prosolia, Inc., Indianapolis, IN). Pixels in the ion images were 200 μm × 200 μm, composed of 10 s or 20 s of data each, for brain and kidney respectively. An individual pixel represents 0.04 mm^2^ of the analyzed surface. Ion images were processed in MSiReader^35^ and had a first order linear interpolation applied, with no normalization and a linear intensity scale. Ion images for individual protein ion charge states were exported as MATLAB .fig files and imported into custom MATLAB software (https://github.com/coopergroup-massspec/sum_matlab_figures). The intensities from each ion image were summed to produce one ion image containing information for multiple protein ion charge states.

### Spectral Deconvolution and Protein Identification

Full-scan and PTCR MS^2^ mass spectra were deconvoluted with UniDec to obtain intact masses.^36^ Top-down identification of proteins was performed with ProSight PC (Thermo) by importing unprocessed MS/MS data and searching against the proteome for *Rattus norvegicus* (UniProt proteome ID: UP000002494), with an intact average mass tolerance of 1000 Da and a sequence ion monoisotopic mass tolerance of 20 ppm. Δ*m* mode was on. Protein matches were initially assessed by the P-score^37^ given by Prosight and the intact mass error. P-score is generally lower where noise may be detected as signals and remain unmatched, generally most applicable to nano-DESI MS*^n^* spectra. As such, manual validation was essential and was performed using the following evidence: assembly and subunit mass accuracy, stoichiometry of the assembly (protein matches that did not form assemblies matching the experimental stoichiometry were eliminated), and detection of sequence ions produced predominantly from cleavage at the C-terminus of aspartic acid residues and the N-terminus of proline residues, which have a high propensity for cleavage in native top-down mass spectrometry by collisional activation.^38^ Ions reported by Prosight were confirmed by the manual investigation of the raw data using TDValidator^39^ (v 1.1, Proteinaceous, Inc., Evanston, IL) or the MS-Product tool in Protein Prospector (v 6.3.1, https://prospector.ucsf.edu/prospector/cgi-bin/msform.cgi?form=msproduct). Where used, TDValidator settings were Max PPM tolerance; 20, Sub ppm tolerance; 3, minimum score; 0.5, S/N cutoff; 3. Unspecified settings were unchanged from defaults. Internal fragments were not assessed. Where acquired, spatial information was also valuable for validating identifications using tissue specificity information from Uniprot entries.

## Results and Discussion

We present a comprehensive analysis of intact endogenous protein assemblies identified directly from a range of tissue types over a broad molecular weight range (up to 145 kDa) by native ambient mass spectrometry (see Table S1, Supporting Information). Three protein assemblies (37.0–66.4 kDa) were identified and imaged in rat brain tissues. These include the cytokine macrophage migration inhibitory factor (MIF, 37.0 kDa), which exists as a homotrimer and the homodimers phosphoglycerate mutase 1 (PGAM1, 57.6 kDa) and malate dehydrogenase 2 (MDH2, 66.4 kDa). Four homodimeric protein assemblies (61.2–94.2 kDa) were identified and imaged in rat kidney tissues. These include omega-amidase (61.2 kDa), MDH2 (66.4 kDa, also seen in the brain), malate dehydrogenase 1 (MDH1, 72.8 kDa), and α-enolase (94.2 kDa). The α-enolase homodimer was observed in its metal-bound form, i.e., with four Mg^2+^ ions (two per subunit). Lastly, two protein assemblies were identified directly from rat liver tissues, the homotrimeric ornithine transcarbamylase (OTC, 108.8 kDa) and homotetrameric lactate dehydrogenase A (LDHA, 145.4 kDa). Putative protein assignments were initially obtained by searching against the protein database with ProSight PC. Potential matches were subsequently validated against the following criteria: the presence of sequence fragments and, in particular, those formed by the cleavage of bonds C-terminal to aspartic acid residues or N-terminal to proline residues (which are known to be favored in native top-down mass spectrometry^38^), observed versus known stoichiometry, and mass accuracy of intact assemblies and subunits. The Supporting Information provides information on Prosight p-scores, % of matched fragment ions that were formed by cleavage C-terminal to Asp or N-terminal to Pro, and MS/MS spectra with fragments indicated.

### Mass Spectrometry Imaging and Identification of Endogenous Protein Assemblies in Brain

**Figure 1:**
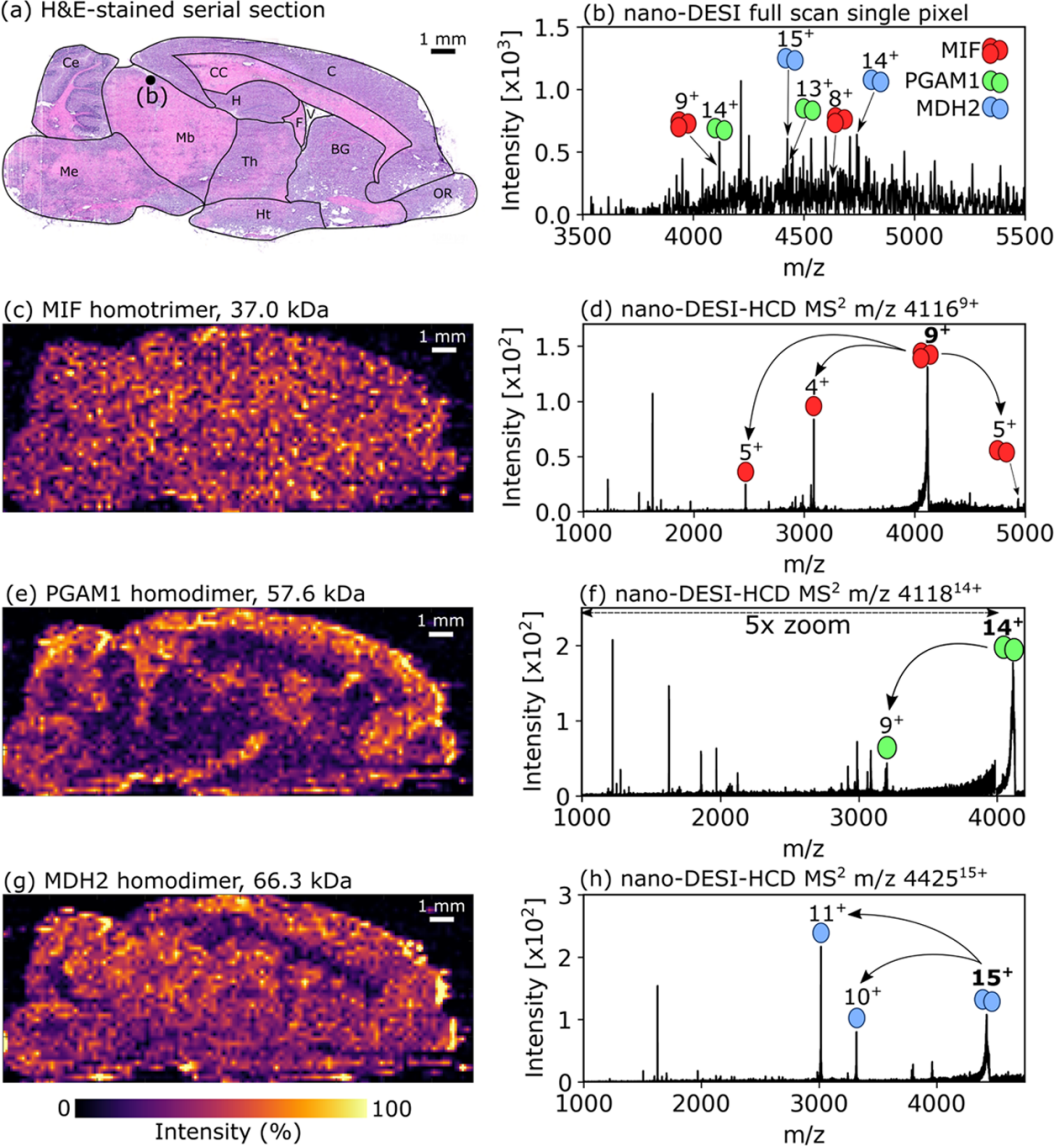
Ion images and HCD MS^2^ spectra indicating subunit dissociation for protein complexes in rat brain. (a) Optical image of H&E-stained serial tissue section. Labels; Ce, cerebellum; C, cerebral cortex; CC, corpus callosum; F, fornix; V, lateral ventricular region; Mb, midbrain; Me, medulla and pons; H, hippocampus; Th, thalamus; Ht, hypothalamus; BG, basal ganglia; and OR, olfactory region. (b) Nano-DESI full-scan mass spectrum representing the pixel marked “(b)” in the optical image. (c, d) Macrophage inhibitory factor homotrimer (37.0 kDa, combination of 9+ and 8+ charge states) showing homogeneous distribution. (e, f) Phosphoglycerate mutase 1 homodimer (57.6 kDa, combination of 14+ and 13+ charge states) and (g, h) MDH2 homodimer (66.3 kDa, combination of 15+ and 14+ charge states). Ion images were produced with a pixel size of 200 μm × 200 μm (0.04 mm^2^), first order linear interpolation, without normalization and with a linear intensity scale. Assigned sequence ions for each protein are included in the Supporting Information.

The spatial distributions of intact protein complexes of molecular weight 37.0–66.4 kDa were mapped throughout a sagittal rat brain section by nano-DESI MSI. A comparison of measured and calculated masses for assemblies and subunits can be found in Table S2, Supporting Information. The optical image of a serial tissue section is shown in Figure 1a. An example mass spectrum corresponding to the pixel marked in Figure 1a is displayed in Figure 1b. The intact mass of intact protein assemblies was determined by high-resolution MS or PTCR (Figure S1, Supporting Information). Figure 1c shows the distribution of MIF (37.0 kDa homotrimer) to be homogeneous throughout brain tissue regions. Nano-DESI-HCD MS^2^ of the 9+ homotrimer ions resulted in a product ion spectrum featuring dissociated monomer (5+, 4+) and dimer (5+) subunits and sequence ions (see Figures 1d, S2 and Table S3, Supporting Information), enabling its identification. The ion image for the homodimer PGAM1 (57.6 kDa) showed high abundance in various regions, especially the cerebral cortex, with lower abundance in the midbrain and corpus callosum (Figure 1e). Nano-DESI-HCD MS^2^ provided sequence and subunit signals, enabling identification of this complex (Figures 1f, S3 and Table S4, Supporting Information). Additionally, the intact mass of the complex is consistent with each subunit featuring a phosphorylated residue. Under a relatively low-energy HCD (approx. 20–25% NCE), a peak corresponds to a neutral loss of ∼80 Da from the intact subunit, suggesting the presence of a phosphorylated tyrosine residue (as indicated in the Uniprot entry),^43,44^ rather than a phosphoserine residue (which would result in a neutral loss of 98 Da). A lack of fragments in the N-terminal region prevented localization of the phosphorylation site. The largely homogeneous spatial distribution of the homodimer MDH2 (66.4 kDa) is shown in Figure 1g, with notable absence in the white matter such as the corpus callosum. In humans, the expression of MDH2 is known in a variety of brain regions including the cerebral cortex and cerebellum (https://www.proteinatlas.org/ENSG00000146701-MDH2/tissue) and that appears to be true here for rats.^45^ Identification of MDH2 homodimer was confirmed by the detection of monomer subunits and sequence ions that populated the nano-DESI-HCD MS^2^ spectrum of *m*/*z* 4425^15+^ (Figures 1h, S4 and Table S5, Supporting Information). Ion images shown in Figure 1 are composite images of multiple charge states. Ion images for individual charge states are shown in Figures S5–S7 in the Supporting Information.

### Mass Spectrometry Imaging and Identification of Endogenous Protein Assemblies in Kidney

**Figure 2:**
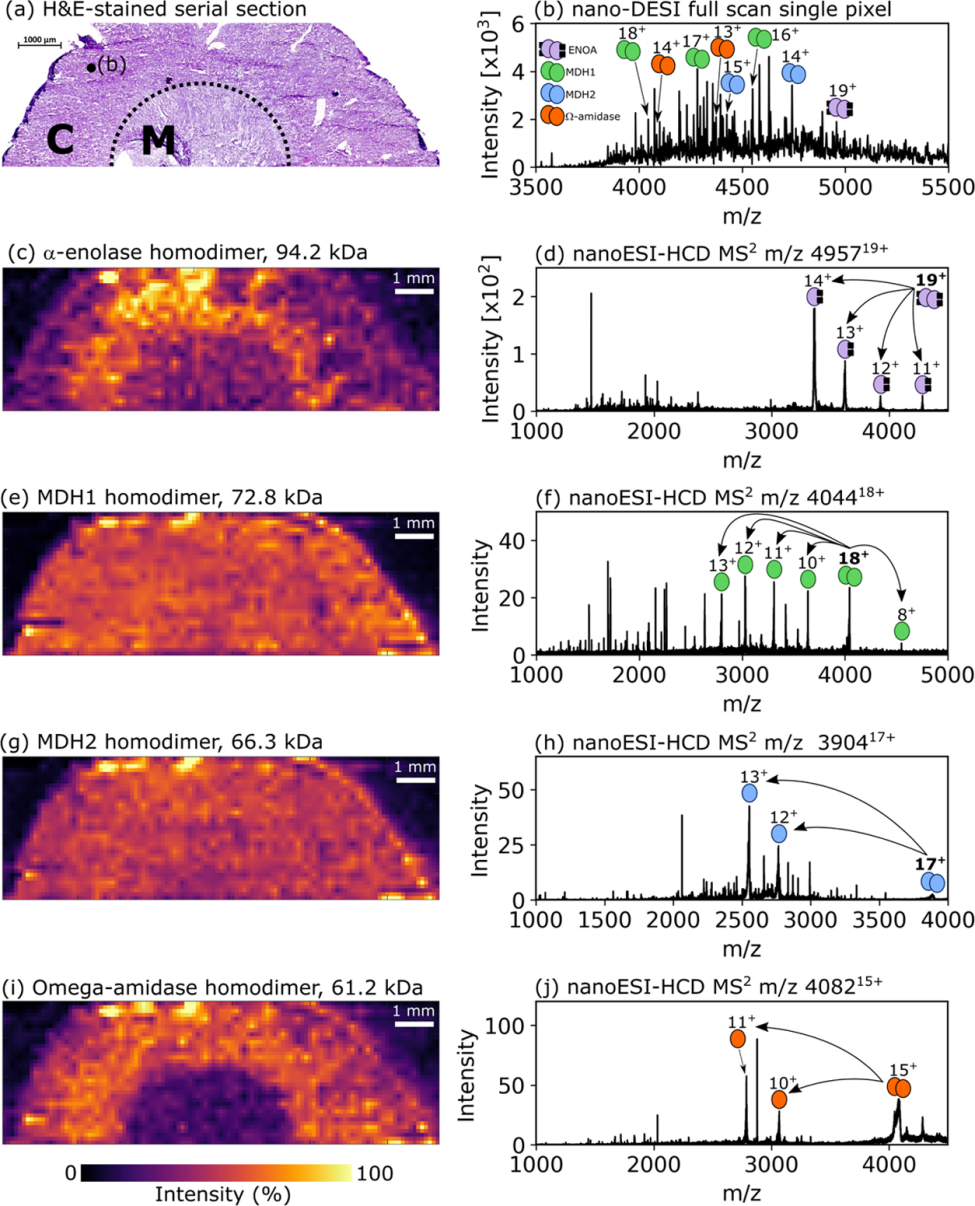
(a) H&E-stained serial section of rat kidney showing cortex (C) and medulla (M) tissues with the approximate boundary marked by the dashed line. (b) Example full-scan mass spectrum for a single nano-DESI pixel in the rat kidney cortex tissue obtained during MSI. The approximate sample location is indicated in panel (a). The mass spectrum is composed of signals averaged over 22 scans and represents an area of 0.04 mm^2^. Ion images show the distributions of intact proteins in the tissue and are paired with nanoESI-HCD spectra indicating subunit dissociation; (c, d) α-enolase homodimer (ENOA, 19+ charge state), 94.2 kDa, cortex tissue; (e, f) malate dehydrogenase 1 (MDH1, combination of 18+, 17+, and 16+ charge states) homodimer, 72.8 kDa, homogeneous; (g, h) MDH2 homodimer (combination of 16+, 15+, and 14+ charge states), 66.6 kDa, homogeneous; (i, j) omega-amidase (combination of 15+ and 14+ charge states) homodimer, 61.2 kDa, cortex tissue. Ion images were produced with a pixel size of 200 μm × 200 μm (0.04 mm^2^), first order linear interpolation, without normalization and with a linear intensity scale. Assigned sequence ions for each protein are included in the Supporting Information.

The spatial distributions of intact protein assemblies with molecular weights 61.2–94.2 kDa were mapped in a sagittal rat kidney tissue section. A comparison of measured and calculated masses for assemblies and subunits can be found in Table S2, Supporting Information. A serial H&E-stained tissue section is shown in Figure 2a. The full-scan mass spectrum for the pixel marked in Figure 2a is shown in Figure 2b. The intact mass of these proteins was determined through PTCR experiments (Figure S8, Supporting Information). The ion image for α-enolase homodimer incorporating two Mg^2+^ ions per subunit (94.2 kDa; Figure 2c) was detected most abundantly in the cortex tissue in agreement with previous immunohistochemical experiments, which found α-enolase to be expressed highly in cortical collecting duct epithelial cells.^46^ The α-enolase dimer is known to bind four Mg^2+^ cofactors under physiological conditions.^47^ While the 20+ charge state of α-enolase was also detected in top-down experiments, intense serum albumin signals at approx. *m*/*z* 4709 (14+ charge state) overlap, so this charge state was excluded from the ion image. Subunit and sequence ions were obtained through HCD (Figures 2d, S9 and Table S6, Supporting Information). The measured average mass of the subunits was consistent with two Mg^2+^ ions being retained during collisional dissociation of the assembly. Both MDH1 (Figures 2e,f, S10 and Table S7, Supporting Information) and MDH2 (Figures 2g,h, S11 and Table S8, Supporting Information) exhibit similar, homogeneous spatial distribution throughout the cortex and medulla tissues. MDH1 mRNA was previously found to be expressed in both proximal tubules (cortex) and medullary collecting tubules.^48^ The ion image for the 61.2 kDa homodimer omega-amidase shows abundance in cortex tissues but is absent in the medulla tissue (Figure 2i), in agreement with antibody staining experiments performed on the human kidney tissue (https://www.proteinatlas.org/ENSG00000114021-NIT2/tissue/kidney).^45^ The nanoESI-HCD MS^2^ spectrum features extensive sequence ion coverage and ions consistent with N-terminal acetylation (Figures 2j, S12 and Table S9, Supporting Information). Ion images for individual charge states of the kidney proteins are shown in Figures S13–S15 in the Supporting Information.

Additional ion images showing the distributions of serum albumin and hemoglobin heterotetramer (Figure S16, Supporting Information) can be found in Figures S17 and S18, Supporting Information, and show protein localization to vascular regions.

### In Situ Top-Down MS of Protein Assemblies Exceeding 100 kDa

**Figure 3:**
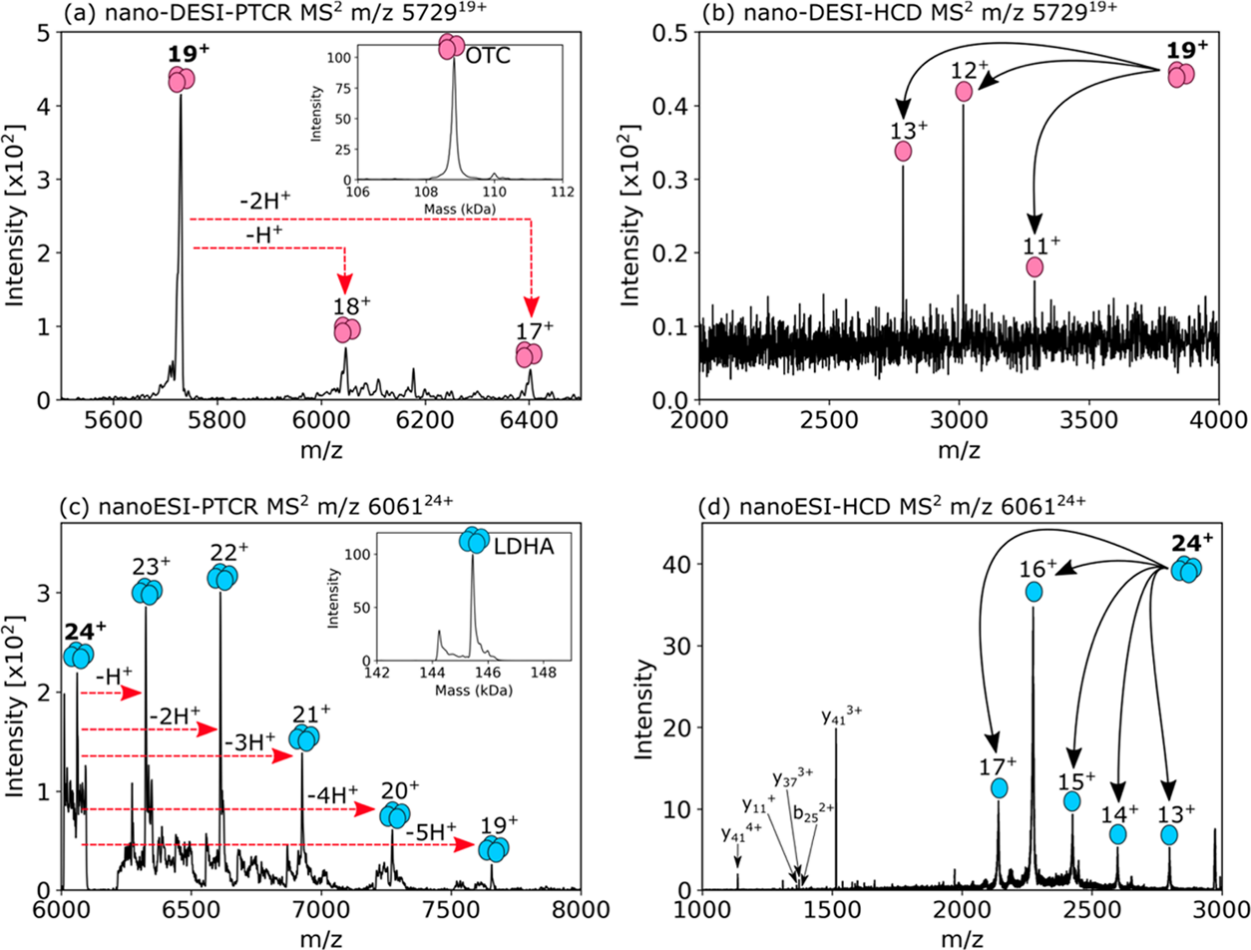
(a) Nano-DESI-PTCR MS^2^ of the intact OTC homotrimer directly from the rat liver tissue. Deconvolution of the mass spectrum confirms the intact mass as 108.8 kDa (inset). (b) Nano-DESI-HCD MS^2^ of the intact OTC homotrimer revealed the subunit mass to be 36.2 kDa. (c) NanoESI-PTCR MS^2^ of the intact LDHA homotetramer (145.4 kDa). (d) NanoESI-HCD MS^2^ of the intact LDHA homotetramer.

Two intact protein assemblies were detected and identified by NAMS of the rat liver tissue. Table S2, Supporting Information, shows their measured and calculated masses. Figure 3a shows the detection of ornithine transcarbamylase (108.8 kDa homotrimer), with the intact mass confirmed by nano-DESI-PTCR MS^2^. The subunit mass (36.2 kDa) was confirmed by collisional dissociation of the 19+ ions of the homotrimer by nano-DESI-HCD MS^2^ (Figure 3b). Sequence information obtained following in-source dissociation of the trimer and subsequent HCD of the monomer subunit (Figure S19 and Table S10, Supporting Information) confirmed the identity of the protein. The measured mass of the subunit ion observed following HCD of the precursor is in excellent agreement (Δ10.2 ppm) with the calculated mass of the subunit; however, there is a mass difference of ∼301 Da between the measured mass of the intact precursor and 3× the measured (and calculated) mass of the subunits. This result suggests the presence of a ligand (or multiple ligands), which dissociates on collisional activation. Ornithine transcarbamylase is known to bind ornithine and carbamoyl phosphate, but no combination of the masses of these species corresponds to the mass difference observed. It is also known to be phosphorylated but, again, this does not account for the mass difference observed.

Figure 3c shows the detection of the homotetramer lactate dehydrogenase A, with the intact mass (145.4 kDa) confirmed by nanoESI-PTCR MS^2^ following LESA microextraction of rat liver. The subunit mass (36.4 kDa) was confirmed by nanoESI-HCD MS^2^ of the 24+ ions of the homotetramer, and the sequence ions detected confirmed the protein identity (Figure 3d). Further confirmation of the protein identity was provided by nanoESI-HCD MS^3^ of the 24+ precursor ions, in which the 16+ monomer subunit was subjected to a further stage of tandem mass spectrometry (Figure S20 and Table S11, Supporting Information). The calculated and measured masses of both the tetramer and the subunits are in good agreement (∼30 ppm). The LDHA homotetramer was also detected with nano-DESI in full-scan mode, albeit with weaker signals and poorer S/N than observed with LESA microextraction and nanoESI, and its presence was confirmed by nano-DESI-PTCR MS^2^ (Figure S21, Supporting Information).

## Conclusions

In this work, we have focused on the detection and identification of endogenous protein assemblies directly from tissues. Eight protein assemblies have been identified, spanning the molecular weight range 37.0–145.4 kDa. The assemblies were all detected in their expected, i.e., functional, stoichiometries, and dimers, trimers, and tetramers were observed. One assembly, the α-enolase dimer, was observed in its metal-bound form. Intact assembly masses were determined either by high-resolution mass spectrometry or by PTCR MS^2^. The latter is shown to be particularly useful as it can confirm the presence and molecular weight of low abundance protein ions without the need for high-resolution mass spectrometry. The upper mass limit detected here, corresponding to the 145.4 kDa LDHA tetramer, exceeds that previously reported by LESA MS by twofold and by nano-DESI by over 100 kDa. These higher molecular weights were accessed by the optimization of ion optics and gas pressures for high *m*/*z* transmission, and it is likely that through ongoing instrumentation and method development, this mass ceiling will be further breached. The low signal intensity for the assemblies exceeding 100 kDa detected here precluded them from nano-DESI MSI experiments, but, again, this challenge is likely to be addressed through further instrument development. Nevertheless, protein assemblies up to 94 kDa were imaged by nano-DESI in brain and kidney tissues. None of the identities of the proteins described in this article were known *a priori*. That is, all of the proteins were identified solely by top-down mass spectrometry, either by standard MS^2^, in which precursor ions were isolated and subjected to HCD, or by pseudo-MS^3^ experiments, in which the assemblies were dissociated in the source region, and the monomeric subunit ions were isolated and fragmented by HCD. Further efforts should be made to improve the number of proteins analyzed within a single imaging experiment, which may be possible using gas-phase enrichment techniques.

## Supporting Information Available

Extended experimental details; summary of measured and theoretical masses of protein assemblies and subunits and associated mass spectra; MS*^n^* spectra and fragment ion tables for all identified proteins; and ion images of individual charge states and additional ion images (PDF)
