# Supplementary material for: Native Ambient Mass Spectrometry Enables Analysis of Intact Endogenous Protein Assemblies up to 145 kDa Directly from Tissue
Source: Anal Chem. 2022 Mar 31;94(14):5608–14. doi: 10.1021/acs.analchem.1c05353 (PMC9008691; doi:10.1021/acs.analchem.1c05353)
Supplement: Supplementary file 1 — ac1c05353_si_001.pdf [file ac1c05353_si_001.pdf]

Supporting Information:

Native ambient mass spectrometry enables analysis of intact endogenous protein assemblies up to 145 kDa directly from tissue.

Oliver J. Hale, James W. Hughes, Emma K. Sisley and Helen J. Cooper*

School of Biosciences, University of Birmingham, Edgbaston B15 2TT, U.K.

TABLE OF CONTENTS

Extended Experimental Details S3
Table S1: Haemotoxylin and eosin staining procedure S4
Table S2: Theoretical and measured mass of identified protein assemblies and subunits S5
Figure S1: Intact mass of protein assemblies in brain S6
Figure S2: MS/MS of MIF S7
Table S3: Sequence ions of MIF S8
Figure S3: MS/MS of PGAM1 S8
Table S4: sequence ions of PGAM1 S9
Figure S4: MS/MS of MDH2 S10
Table S5: sequence ions for MDH2 S10
Figure S5: ion images for MIF S11
Figure S6: ion images for PGAM1 S11
Figure S7: ion images for MDH2 S12
Figure S8: Intact mass of protein assemblies in kidney S12
Figure S9: MS/MS of α-enolase S13
Table S6: sequence ions for a-enolase S14
Figure S10: MS/MS of MDH1 S15
Table S7: sequence ions for MDH1 S16
Figure S11: MS/MS of MDH2 S17
Table S8: sequence ions of MDH2 S18
Figure S12: MS/MS of omega-amidase S20
Table S9: sequence ions of omega-amidase S21
Figure S13: ion images for MDH1 S22
Figure S14: ion images for MDH2 S22
Figure S15: ion images for omega-amidase S23
Figure S16: native mass spectrum obtained from a blood vessel S23
Figure S17: ion images for serum albumin S24
Figure S18: ion images for hemoglobin tetramer S24
Figure S19: MS/MS of OTC S25
Table S10: sequence ions for OTC S25

Figure S20: MS^3 of LDHA S26
Table S11: sequence ions for LDHA S27
Figure S21: nano-DESI analysis of intact LDHA S27
References S28

EXPERIMENTAL DETAILS

Nano-DESI

An XYZ-stage (Zaber Technologies Inc., Vancouver, Canada) was mounted at the mass spectrometer inlet. Solvent and sampling capillaries were flame-pulled from fused silica tubing (O.D. 275 μm, I.D. 75 μm prior to modification) and cut to a final outer diameter of approx. 100 μm. The sampling capillary was positioned approx. 0.5 mm within the mass spectrometer inlet to aspirate solvent with the inlet vacuum. The exit of the sampling capillary was not flame-pulled but had the coating removed. Solvent was delivered through the solvent capillary by a 10 mL gas-tight syringe (Hamilton, Reno, NV) at a flow rate of 1.9-2.0 μL/min and a liquid junction was formed between the two capillaries. High voltage was provided by a wire from the mass spectrometer power supply connected directly to the syringe needle and optimized for sensitivity and spray stability (typically between 0.8 and 1.5 kV). Electrospray optimization was performed on the protonated detergent ion signal (C8E4: m/z 307.24) to achieve an RSD% of <10% and a spray current between 0.1 and 0.5 μA. Each nano-DESI probe has slightly different characteristics and will optimize differently but this method provided consistent performance for native protein analysis with the solvent systems used.

Stage movement was controlled directly by Zaber Control (Zaber Technologies Inc.) or automated by custom software written in LabVIEW (NI, Austin, Texas). *In situ* intact protein profiling and top-down mass spectrometry analyses were performed by positioning thin tissue sections underneath the nano-DESI probe and scanning the probe across the surface at between 1 and 20 μm/s. A relay connected to an Arduino Uno v3 microcontroller triggered contact closure to signal the start of a new line scan in MSI experiments.

LESA microextraction and nanoelectrospray ionization

Proteins were extracted from tissue by LESA microextraction using a Triversa Nanomate robot (Advion Biosciences, Ithaca, NY) and extraction solvents comprising ammonium acetate (200 mM) containing detergent (specified with relevant data). A 96 well plate was cut in half and loaded into the well plate mount. The glass slide containing the thaw mounted tissue was secured in the remaining space of the well plate mount using adhesive putty. A method was defined in the advanced user interface (AUI) of the ChipSoft software whereby 5 µL of the sampling solvent was aspirated from one of the wells of the well plate into a pipette tip. The pipette was positioned over the tissue and the pipette was brought in to contact with the tissue surface ("contact" LESA)[1]. 2 µL solvent was dispensed and the tissue-liquid junction was maintained for 30 seconds including 10 dispense-respirate cycles before the solvent (2 µL) was re-aspirated into the pipette tip. The pipette was repositioned above a clean well

and the sample extract was deposited (5 µL). 4 µL of the solvent extract was transferred into a nanoESI emitter by use of a manual pipette fitted with a gel loading tip.

Haemotoxylin and eosin staining procedure

Haemotoxylin and eosin staining was performed as described in Table S1. Thaw mounted tissue sections were sequentially submerged in solvent baths containing the described solvent and for the time period listed. Where a repeat submersion was required, a fresh bath of solvent was used. Tissue was mounted in DPX and a coverslip was applied before optical imaging.

Table S1: Haemotoxylin and eosin staining procedure.

**Solvent Bath**	**Time (min)**	**Repeat**
Water	2	1
Haemotoxylin Harris	4	0
Water	2	1
Acid Alcoholc	0.5	0
Water	2	1
Scott's tap Water Substitute	0.5	0
Water	2	1
Eosin	1	0
Water	2	1
Industrial denatured alcohol	2	3
Xylene	2	2

Table S2: Theoretical and measured average mass of identified protein assemblies and subunits.

	Protein	Uniprot entry	Assembly Stoichiometry	PTMs	Mass (assembly) (Da)	Theoretical mass (assembly) (Da)	Mass difference (ppm)	Measured mass (subunit) (Da)	Theoretical mass (subunit) (Da)	Mass difference (ppm)
**Brain**	MIF	P30904	Homotrimer	N/A	37038.5	37038.5	0.9	12346.1	12346.2	-9.0
	PGAM1	P25113	Homodimer	N-term acetylation, Y-phosphorylation	57649.6	57646.1	61.3	28822.6	28823.1	-14.3
	MDH2	P04636	Homodimer	N/A	66366.2	66365.4	13.0	33182.3	33182.7	-12.3
**Kidney**	α-enolase (apo)	P04764	Monomer	N-term acetylation	N/A	N/A	N/A	47039.2	47038.7	11.1
	α-enolase (holo)	P04764	Homodimer + 4x Mg^{2+}	N-term acetylation	94176.9	94175.7	12.8	47086.1	47087.9	-36.8
	MDH1	O88989	Homodimer	N-term acetylation	72789.6	72787.9	23.2	36393.7	36394.0	-7.8
	MDH2	P04636	Homodimer	N/A	66366.0	66365.4	10.0	33183.4	33182.7	21.6
	Ω-amidase	O88989	Homodimer	N-term acetylation	61224.7	61224.2	8.7	30610.3	30612.1	-57.4
**Liver**	OTC	P00481	Homotrimer	N/A	108831.7	108529.9	2773.5	36177.0	36176.6	10.2
	LDHA	P04642	Homotetramer	N-term acetylation	145449.0	145445.3	25.6	36360.2	36361.3	-30.6

Rat brain

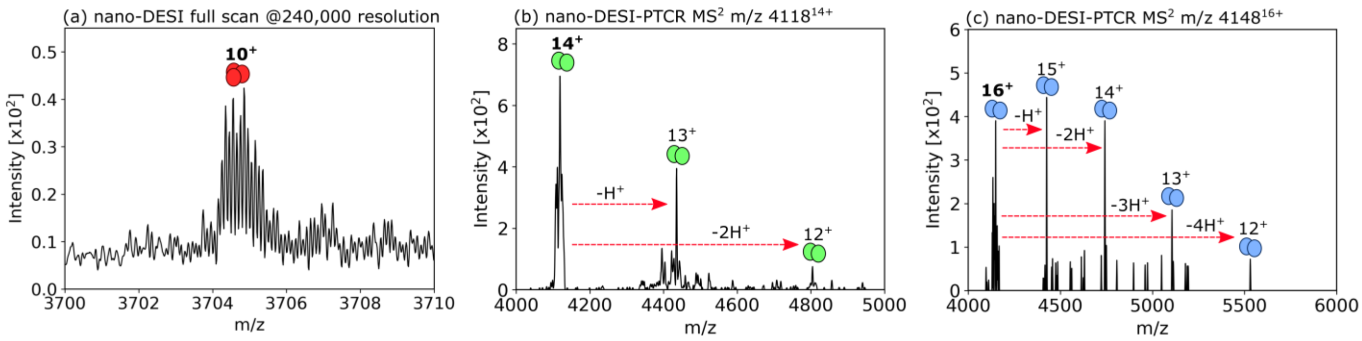

Figure S1: Determination of the intact mass of protein assemblies in rat brain. (a) nano-DESI full scan MS spectrum at a nominal resolution of 240,000 (m/z 200) showing isotopically resolved signals for MIF in the 10+ charge state (approx. 37.0 kDa). ((b) nano-DESI-PTCR MS2 spectrum for m/z 4118 indicating precursor ions in the 14+ charge state (approx. 57.6 kDa). (c) nano-DESI-PTCR MS2 spectrum for m/z 4148 indicating precursor ions in the 16+ charge state (approx. 66.3 kDa). PTCR spectra were acquired at orbitrap resolution = 7500 at m/z 200 using a 200 mM ammonium acetate + 0.5x CMC C8E4 solvent system.

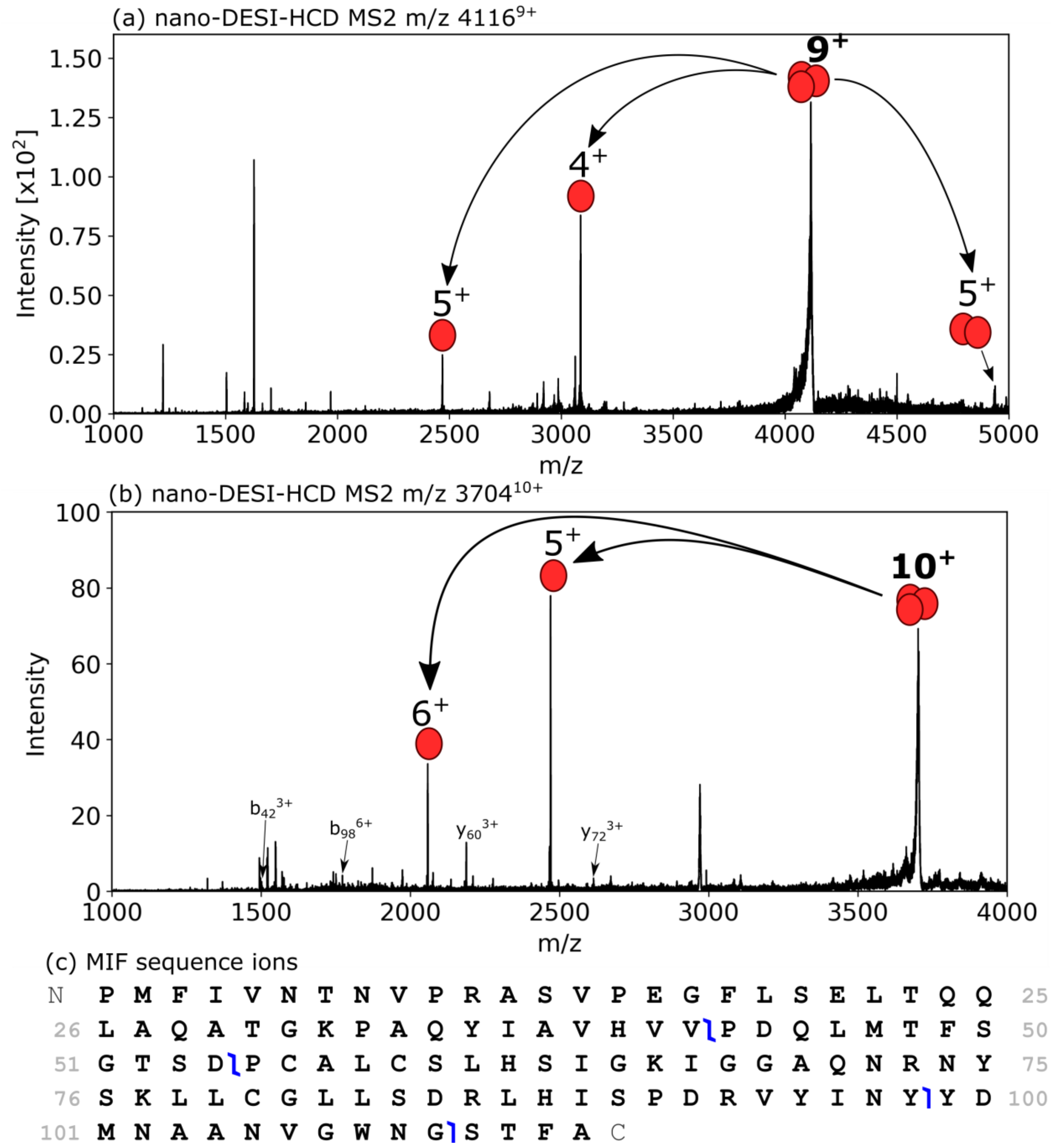

Figure S2: (a) nano-DESI-HCD MS2 of the 9+ (NCE = 45 – 46%) and (b) 10+ (NCE = 40%) charge state of macrophage inhibitory factor (MIF) homotrimer. Sequence ions are indicated for the 10+ charge state because of co-isolation of unrelated protein ion signals in the ion trap window for the 9+ charge state. Subunit ions confirm the assembly stoichiometry as homotrimeric. Mass spectra acquired on the Orbitrap Eclipse with an orbitrap resolution of 240,000 at m/z 200 using a 200 mM ammonium acetate + 0.5x CMC C8E4 solvent system. (c) Sequence ions assigned with a mass tolerance of 20 ppm. P-score = 0.054 with 4 of 6 ions explained by cleavages at D and P residues.

Table S3: Sequence ions of MIF.

Name	Theoretical Mass (Da)	Observed Mass (Da)	Mass Difference (Da)	Mass Difference (ppm)
**b42**	4502.3784	4502.3108	-0.0676	-15.0
**b54**	5781.9174	5781.8294	-0.0880	-15.2
**b98**	10621.4061	10621.5618	0.1557	14.7
**b110**	11913.9305	11914.0891	0.1586	13.3
**y60**	6556.2089	6556.1716	-0.0373	-5.7
**y72**	7835.7479	7835.6321	-0.1158	-14.8

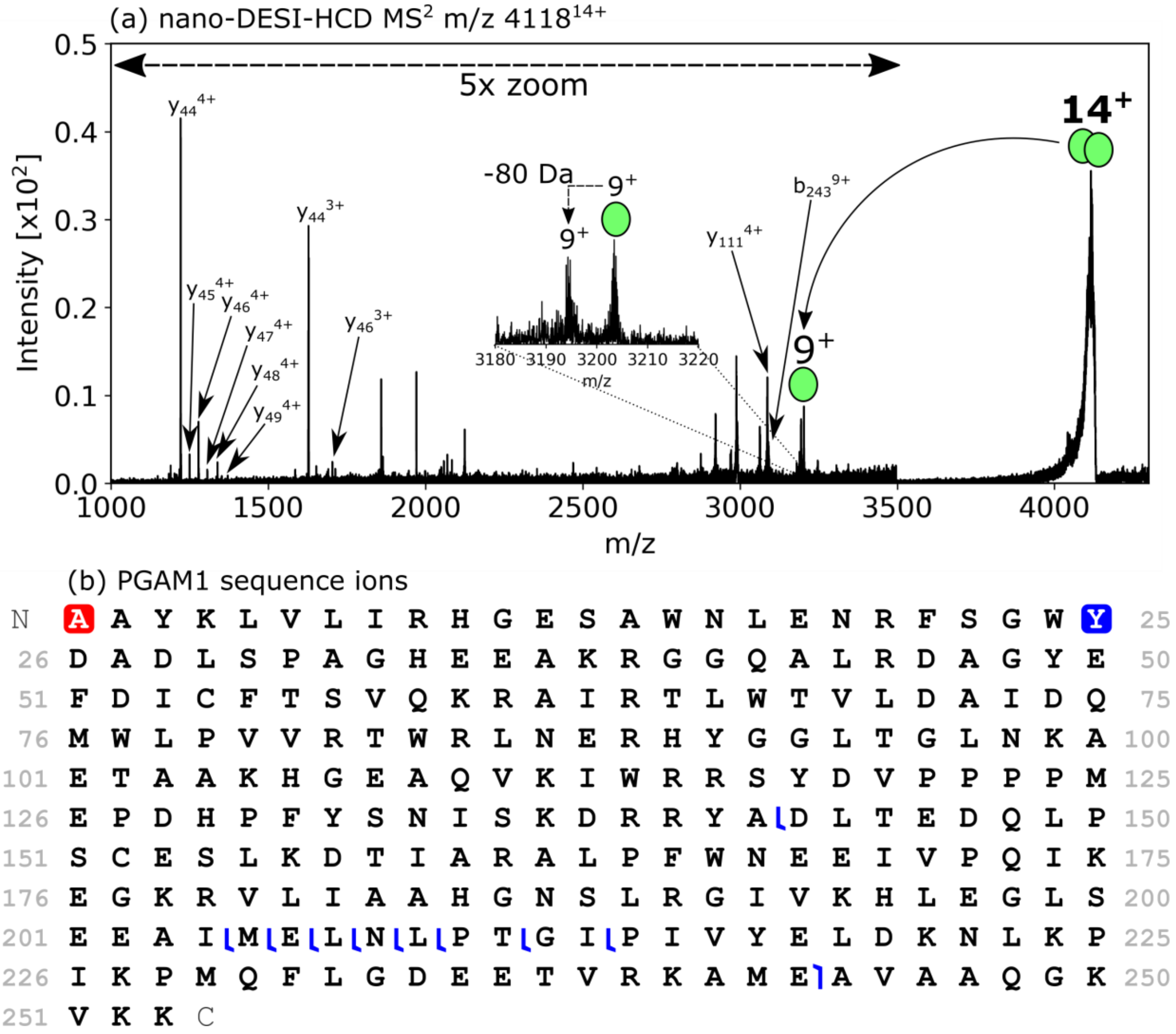

Figure S3: (a) nano-DESI-HCD MS^2 spectrum for phosphoglycerate mutase obtained with NCE = 20 – 25%. The intact mass is consistent with N-terminal acetylation (red highlight) and a single phosphorylation (blue highlight) situated towards the N-terminus. Spectrum acquired on the Orbitrap Eclipse with an orbitrap resolution of 240,000 at m/z 200 using a 200 mM ammonium acetate + 0.5x CMC C8E4 solvent system. (b) Sequence ions for PGAM1 assigned with a mass tolerance of 20 ppm. P-score = 0.067. 2 of 10 ions are explained by cleavages N-terminally to P-residues. The region I204 – P214 shows comprehensive cleavage, with the y44 ion showing as especially abundant. The precise phosphorylated residue was not determined due to the absence of identifiable b-ion signals, but is included on the Y-residue (highlighted in blue) as per annotations on Uniprot (P25113) due to the prevalence of neutral loss = 80 Da rather than 98 Da.

Table S4: Sequence ions of PGAM1

Name	Theoretical Mass (Da)	Observed Mass (Da)	Mass Difference (Da)	Mass Difference (ppm)
**y40**	4511.4647	4511.4203	-0.0444	-9.9
**y42**	4681.5702	4681.5313	-0.0390	-8.3
**y44**	4879.6707	4879.6038	-0.0669	-13.7
**y45**	4992.7547	4992.7020	-0.0527	-10.6
**y46**	5106.7977	5106.7352	-0.0624	-12.2
**y47**	5219.8817	5219.8276	-0.0541	-10.4
**y48**	5348.9243	5348.8898	-0.0345	-6.4
**y49**	5479.9648	5479.9110	-0.0538	-9.8
**y111**	12337.3724	12337.5840	0.2116	-17.1
**b243**	27806.1813	3090.6055	-0.2052	7.4

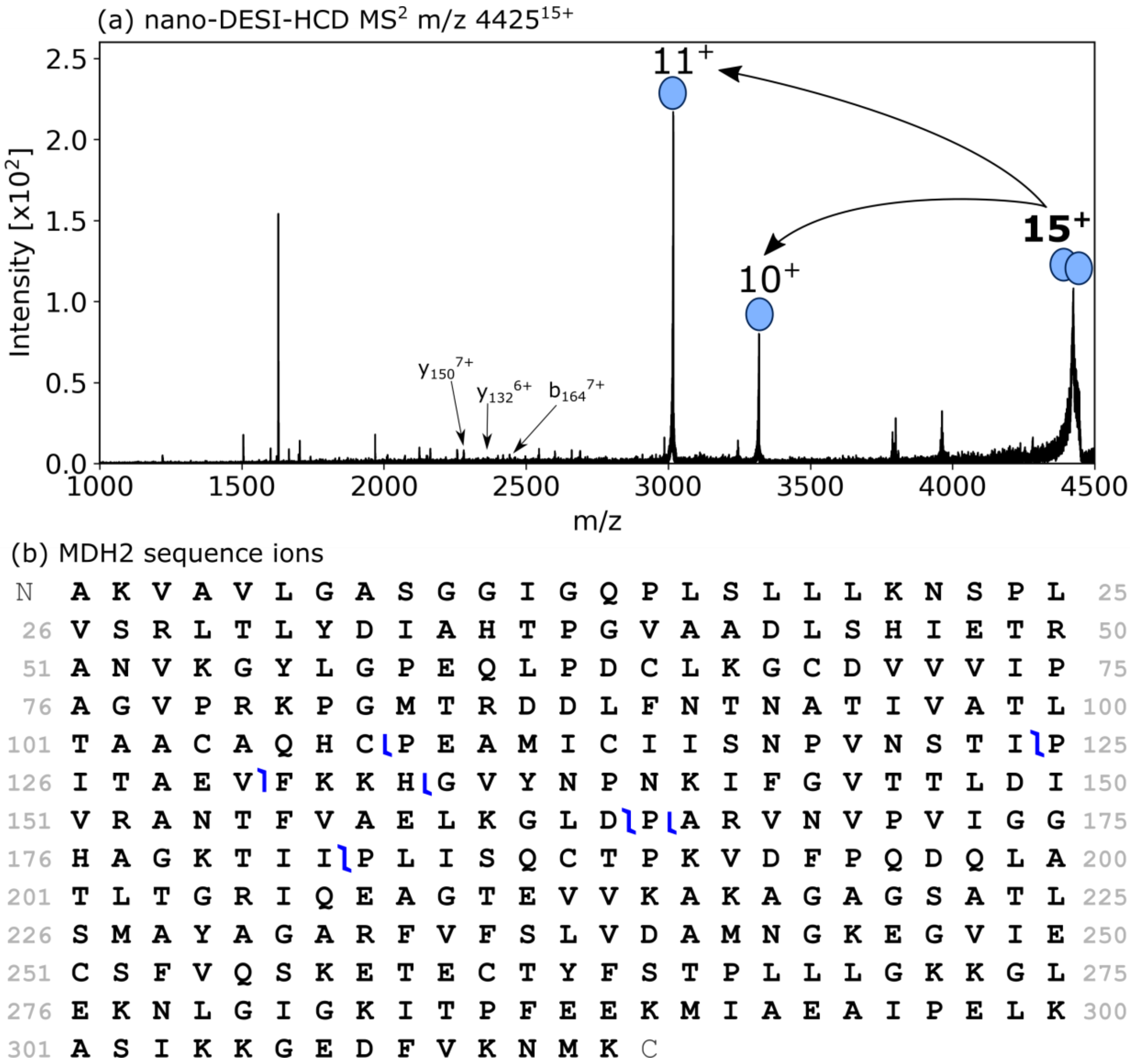

Figure S4: (a) nano-DESI-HCD MS^2 spectrum of m/z 4425^{15+}, MDH2 homodimer, showing subunit ions in 11+ and 10+ charge states (NCE = 50%) and confirming assembly stoichiometry as homodimeric. Spectrum acquired on the Orbitrap Eclipse with an orbitrap resolution of 240,000 at m/z 200 using a 200 mM ammonium acetate + 0.5x CMC C8E4 solvent system. (b) Sequence ions assigned with a mass tolerance of 20 ppm. P-score = 0.13. 7 of 9 ions are explained by cleavage at D or P residues. There is some fragment ion overlap with the MDH2 identified in the kidney by nESI (Figure S11).

Table S5: sequence ions for MDH2.

Name	Theoretical Mass (Da)	Observed Mass (Da)	Mass Difference (Da)	Mass Difference (ppm)
**b124**	12816.7017	12816.6559	-0.0458	-3.6
**b130**	13427.0343	13426.9893	-0.0450	-3.3
**b164**	17213.0963	17213.0173	-0.0790	-4.6
**b182**	18993.1434	18993.1327	-0.0107	-0.6
**y132**	14168.3591	14168.2997	-0.0594	-4.2
**y150**	15948.4062	15948.3493	-0.0569	-3.6
**y180**	19194.1510	19194.0491	-0.1019	-5.3
**y190**	20344.8008	20344.7192	-0.0816	-4.0
**y206**	22027.6380	22027.5247	-0.1133	-5.1

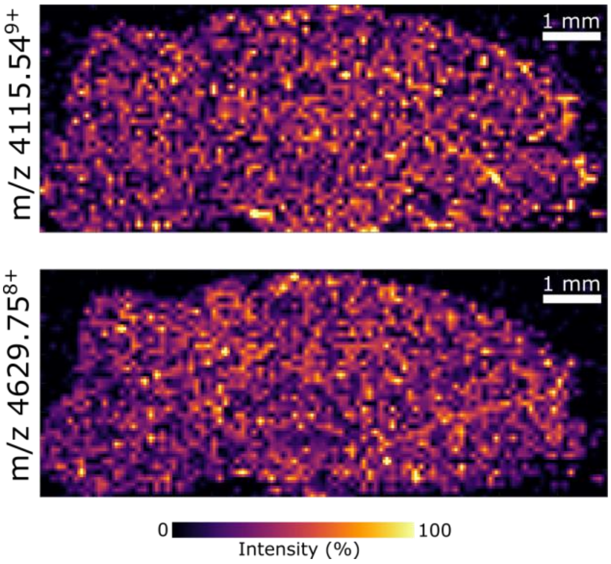

Figure S5: Ion images for 9+ and 8+ charge states of MIF showing homogenous distribution.

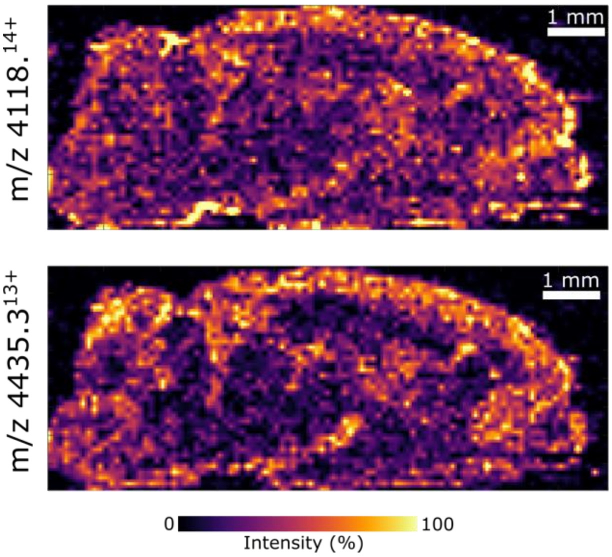

Figure S6: Ion images for 14+ and 13+ charge states of PGAM1 showing distribution in regions such as the cerebral cortex.

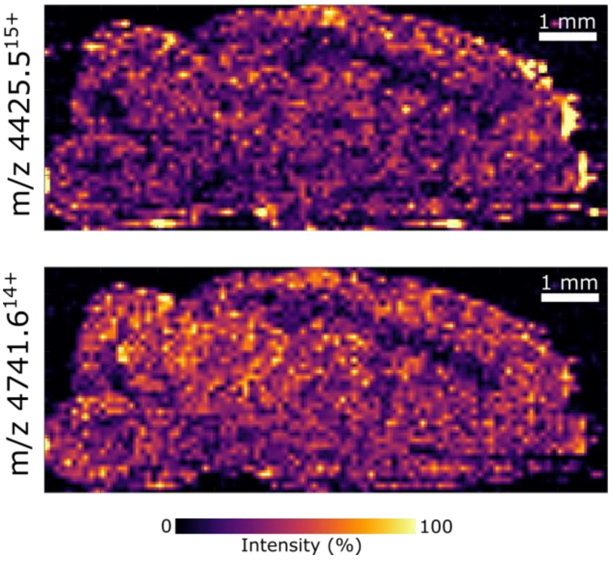

Figure S7: Ion images for 15+ and 14+ charge states of MDH2 showing distribution amongst grey matter.

Rat kidney:

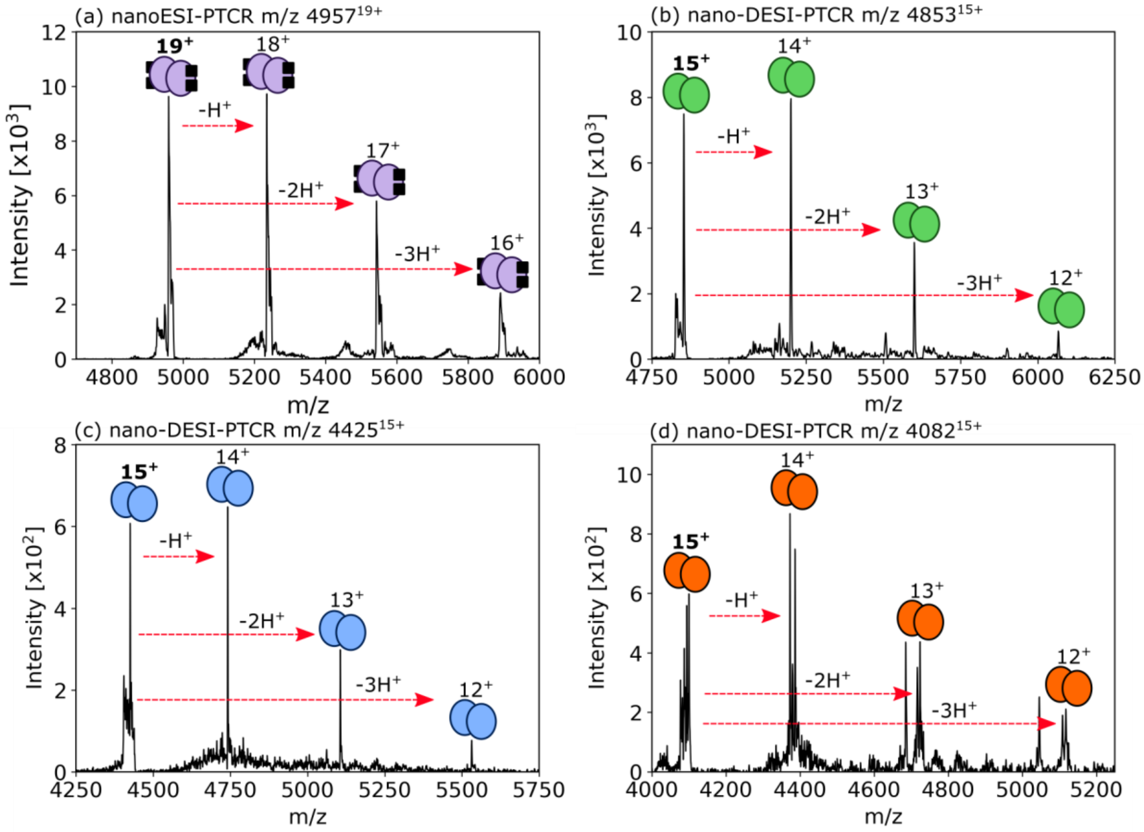

Figure S8: Determination of intact mass of kidney protein assemblies. (a) nanoESI-PTCR MS^2 spectrum of m/z 4957 indicating precursor ions in the 19+ charge state. (b) nano-DESI-PTCR MS^2 spectrum of m/z 4853 indicating precursor ions in the 15+ charge state (approx. 72.8 kDa). (c) nano-DESI-PTCR MS2 spectrum for m/z 4425 indicating precursor ions in the 15+ charge state (approx. 66.3 kDa). (d) nano-DESI-PTCR MS2 spectrum for m/z 4082 indicating precursor ions in the 15+ charge state (approx. 61.3 kDa). PTCR spectra were acquired with an orbitrap resolution of 7500 at m/z 200.

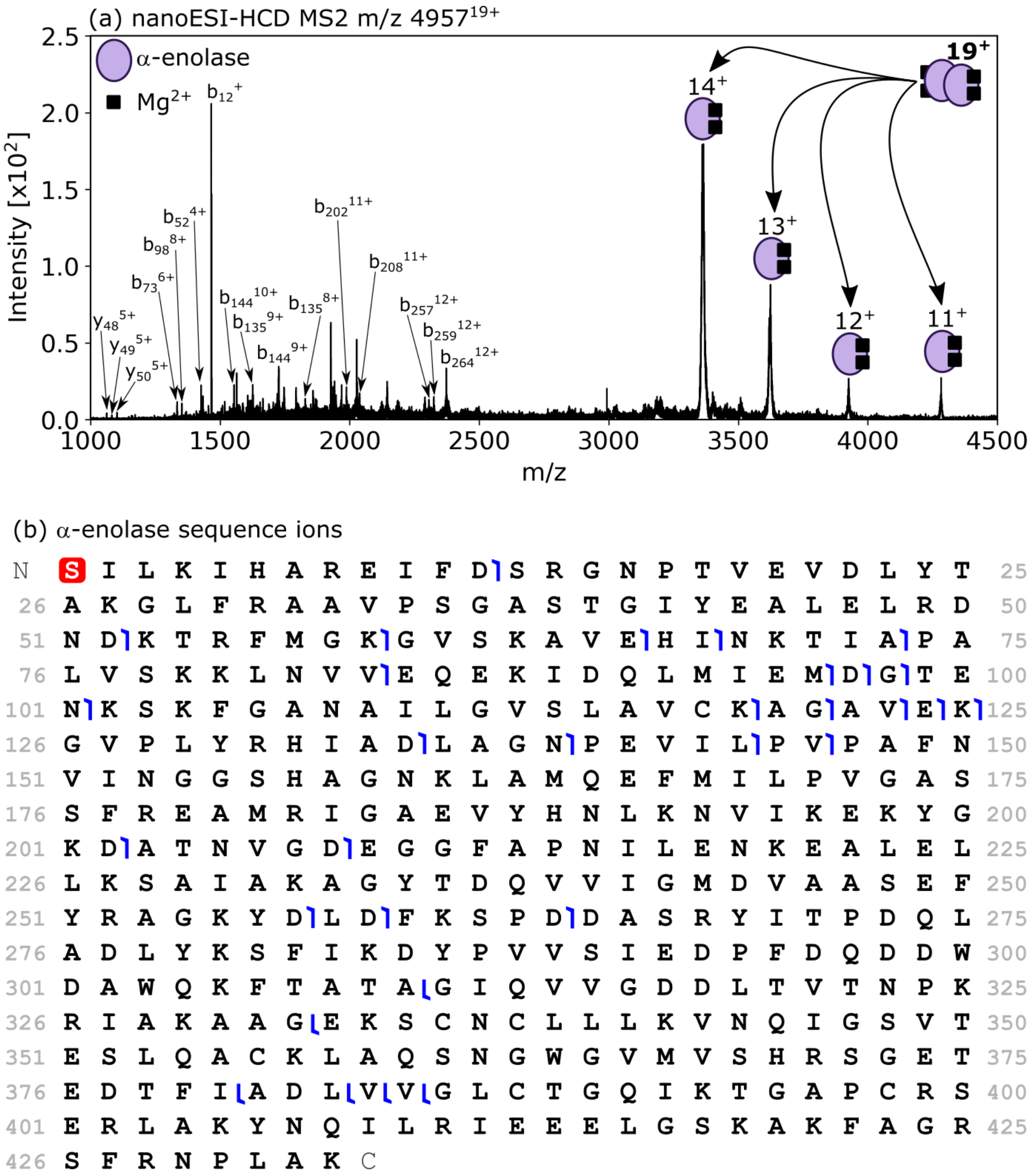

Figure S9: (a) nanoESI-HCD MS^2 spectrum of α-enolase homodimer (NCE = 40%) showing subunits and sequence ions. The subunit ions are consistent with the intact assembly being homodimeric. Spectrum acquired on the Orbitrap Eclipse using 200 mM aqueous ammonium acetate + 2x CMC LDAO and an orbitrap resolution of 120,000 at m/z 200. (b) sequence ions of N-terminally acetylated alpha-enolase assigned with a mass tolerance of 20 ppm. P-score = 0.00043. 13 of 31 ions are explained by cleavages at D and P residues.

Table S6: sequence ions for α-enolase

Name	Theoretical Mass (Da)	Observed Mass (Da)	Mass Difference (Da)	Mass Difference (ppm)
**b12**	1464.8089	1464.7992	-0.0096	-6.6
**b52**	5698.9382	5698.9239	-0.0143	-2.5
**b59**	6547.4073	6547.3621	-0.0452	-6.9
**b66**	7217.7723	7217.6770	-0.0953	-13.2
**b68**	7467.9153	7467.8704	-0.0449	-6.0
**b73**	7995.2220	7995.1307	-0.0913	-11.4
**b84**	9143.9501	9143.8183	-0.1318	-14.4
**b96**	10631.6501	10631.5405	-0.1096	-10.3
**b97**	10746.6770	10746.5348	-0.1423	-13.2
**b98**	10813.0164	10813.0884	-0.0720	6.7
**b101**	11147.8317	11147.6309	-0.2007	-18.0
**b119**	12934.8444	12934.7204	-0.1240	-9.6
**b121**	13062.9030	13062.7301	-0.1729	-13.2
**b123**	13233.0085	13232.8609	-0.1476	-11.2
**b124**	13362.0511	13361.8760	-0.1751	-13.1
**b125**	13490.1460	13489.9080	-0.2380	-17.6
**b135**	14622.2267	14622.1430	0.0837	-5.7
**b139**	14966.9298	14966.8227	-0.1071	-7.2
**b144**	15518.2616	15518.1042	-0.1575	-10.1
**b146**	15714.3828	15714.2580	-0.1248	-7.9
**b202**	21855.3286	21855.1922	0.1364	-6.2
**b208**	22399.7612	22399.4078	-0.3534	-15.8
**b257**	27657.3870	27657.4242	-0.0372	1.3
**b259**	27885.4986	27885.5358	-0.0372	1.3
**b264**	28459.7730	28459.8066	-0.0336	1.2
**y48**	5304.8451	5304.7491	-0.0960	-18.1
**y49**	5409.7373	5409.7063	0.0310	-5.7
**y50**	5508.8058	5508.7558	0.0500	-9.1
**y53**	5802.1301	5802.0912	-0.0389	-6.7
**y101**	10993.6499	10993.7733	0.1233	11.2
**y123**	13197.8776	13197.7840	-0.0936	-7.1

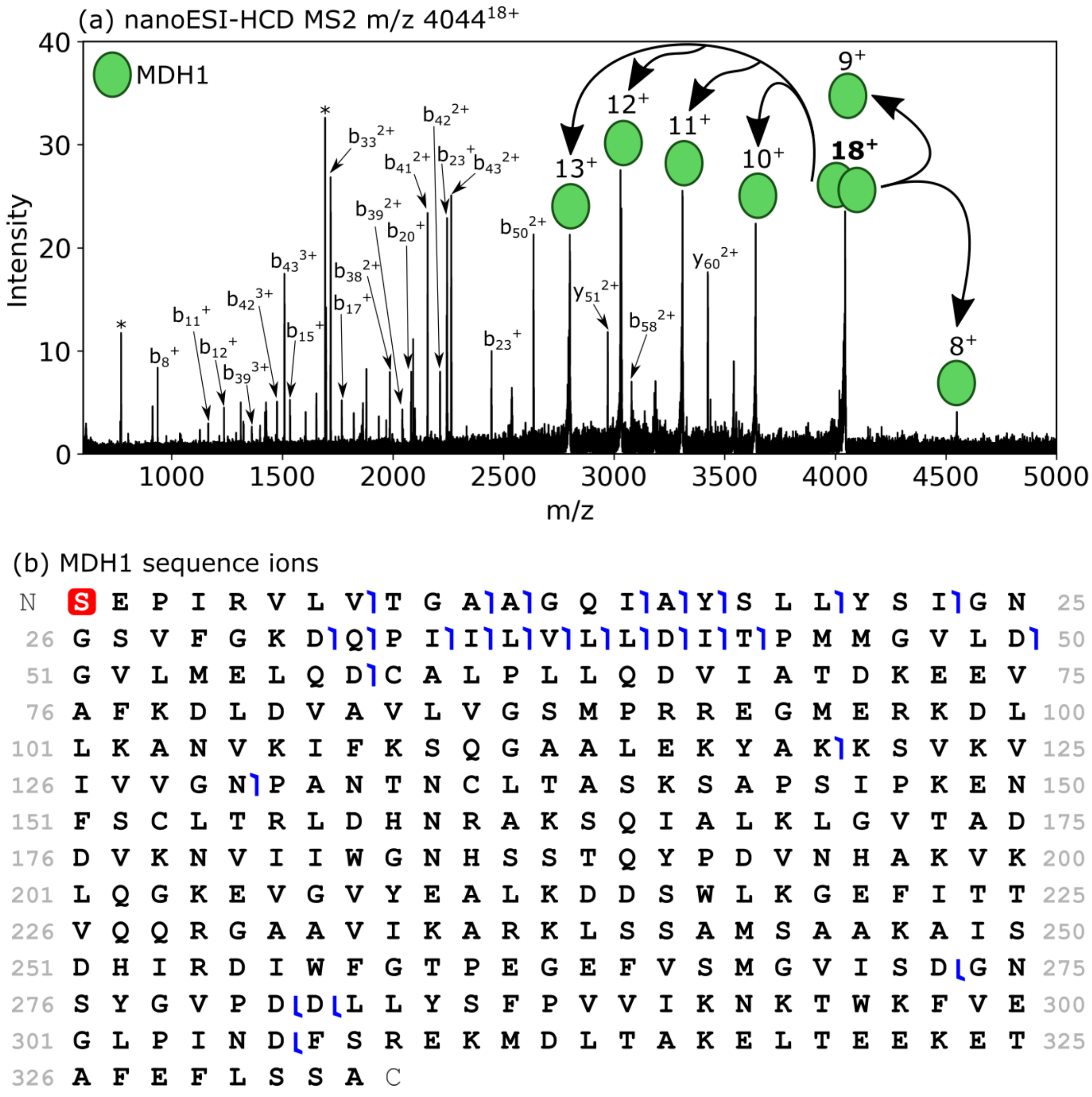

Figure S10: (a) nanoESI-HCD MS^2 spectrum of MDH1 homodimer showing subunits and sequence ions. Subunit ions confirm the assembly stoichiometry as homodimeric. The 9+ monomer signal overlaps the 18+ precursor ion signal. Spectrum acquired on the QE-HF with an orbitrap resolution of 120,000 at m/z 200. (b) sequence ions of N-terminally acetylated MDH1 assigned with a mass tolerance of 20 ppm. P-score = 3e-15. 11 of 27 ions are explained by cleavage at D or P residues. Data were acquired on the QE-HF, NCE= 30%, 200 mM aqueous ammonium acetate + 2x CMC LDAO.

Table S7: sequence ions for MDH1

Name	Theoretical Mass (Da)	Observed Mass (Da)	Mass Difference (Da)	Mass Difference (ppm)
**b8**	935.5440	935.5474	0.0034	3.6
**b11**	1164.6505	1164.6491	0.0014	-1.2
**b12**	1235.6874	1235.6807	-0.0066	-5.4
**b15**	1533.8515	1533.8561	0.0047	3.0
**b16**	1604.8886	1604.8936	0.0051	3.2
**b17**	1767.9519	1767.9542	0.0023	1.3
**b20**	2081.1520	2081.1421	-0.0100	-4.8
**b23**	2444.3315	2444.2989	-0.0326	-13.3
**b32**	3305.7295	3305.7335	0.0039	1.2
**b33**	3433.7881	3433.7672	-0.0209	-6.1
**b35**	3643.9249	3643.9112	-0.0137	-3.8
**b36**	3757.0090	3756.9844	-0.0246	-6.6
**b37**	3870.0930	3870.0817	-0.0114	-2.9
**b38**	3969.1615	3969.1603	-0.0011	-0.3
**b39**	4082.2455	4082.2462	0.0007	0.2
**b40**	4195.3296	4195.3127	-0.0169	-4.0
**b41**	4310.3565	4310.3656	0.0091	2.1
**b42**	4423.4406	4423.4107	-0.0299	-6.8
**b43**	4524.4883	4524.4536	-0.0347	-7.7
**b50**	5267.8229	5267.7479	-0.0750	-14.2
**b58**	6153.2495	6153.1801	-0.0694	-11.3
**b120**	12992.9401	12992.9429	0.0028	0.2
**b130**	14016.5860	14016.6045	-0.0185	1.3
**y27**	3136.5274	3136.5137	-0.0137	-4.4
**y51**	5938.0793	5938.0832	0.0039	0.7
**y52**	6053.1063	6053.0942	-0.0121	-2.0
**y60**	6842.4356	6842.3604	-0.0752	-11.0

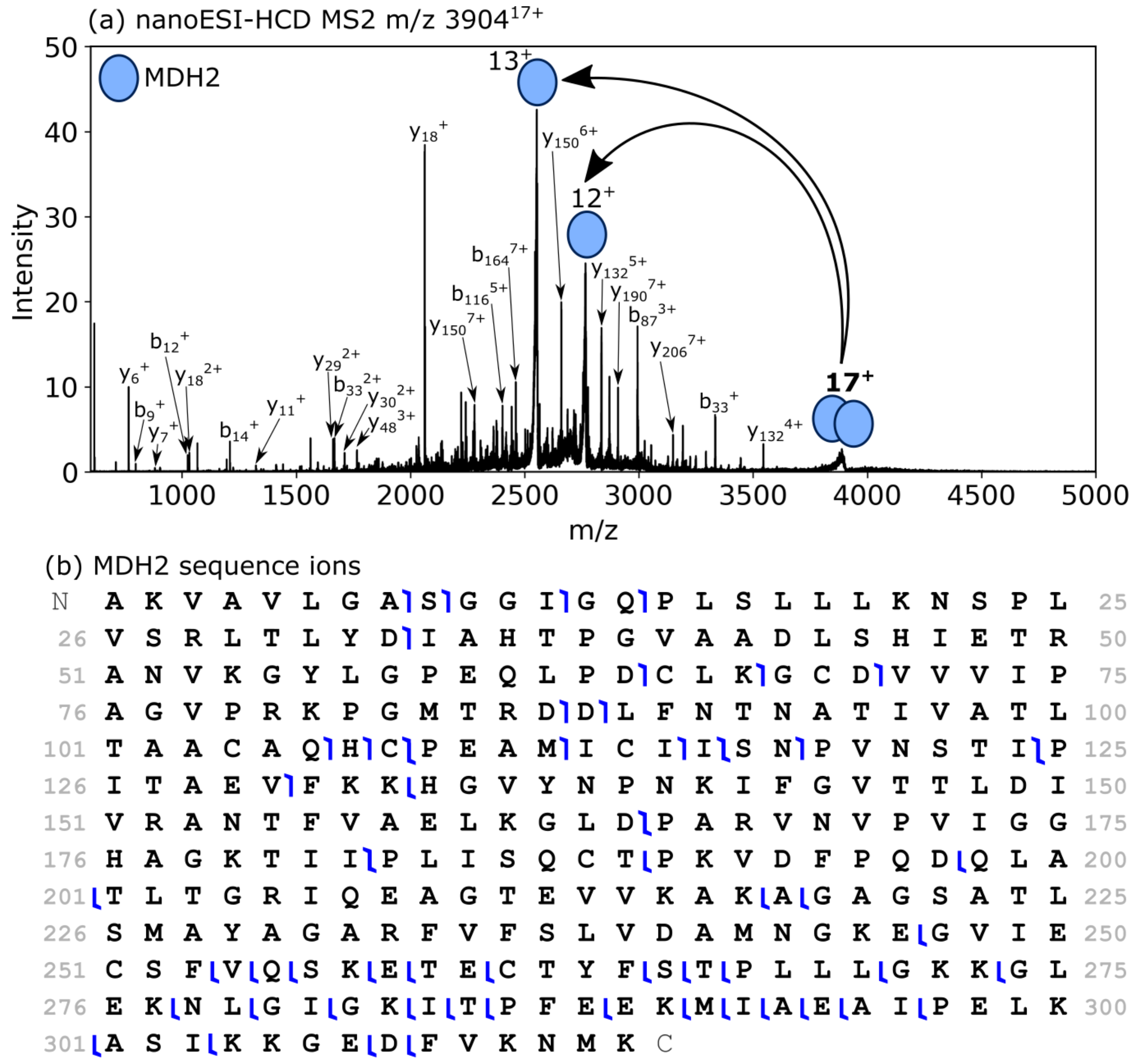

Figure S11: (a) nanoESI-HCD MS² spectrum of MDH2 showing subunits and sequence ion signals. Subunit ions confirm the assembly stoichiometry as homodimeric. Spectrum acquired on the QE-HF with an orbitrap resolution of 120,000 at m/z 200. (b) sequence ions of MDH2 assigned with a mass tolerance of 20 ppm. P-score = 2.8e-44. 21 of 61 ions are explained by cleavage at D or P residues. Data were acquired on the QE-HF, NCE = 26%, in 200 mM aqueous ammonium acetate + 2x CMC LDAO.

Table S8: sequence ions for MDH2

Name	Theoretical Mass (Da)	Observed Mass (Da)	Mass Difference (Da)	Mass Difference (ppm)
**b8**	709.4486	709.4516	0.0030	4.2
**b9**	796.4807	796.4837	0.0031	3.9
**b12**	1023.6076	1023.6007	-0.0070	-6.8
**b14**	1208.6877	1208.6928	0.0051	4.2
**b33**	3331.9231	3331.8772	-0.0459	-13.8
**b64**	6582.5965	6582.6395	0.0431	6.5
**b67**	6926.7847	6926.7558	-0.0289	-4.2
**b70**	7201.8423	7201.7894	-0.0529	-7.3
**b87**	8974.8506	8974.8402	-0.0104	-1.2
**b88**	9089.8775	9089.8829	0.0054	0.6
**b106**	10893.7964	10893.7466	-0.0498	-4.6
**b107**	11030.8553	11030.8523	-0.0030	-0.3
**b108**	11133.8645	11133.7957	-0.0688	-6.2
**b112**	11562.0375	11561.9269	-0.1105	-9.6
**b115**	11891.2148	11891.1617	-0.0531	-4.5
**b116**	12004.2988	12004.2738	-0.0250	-2.1
**b118**	12205.3738	12205.3202	-0.0536	-4.4
**b124**	12816.7017	12816.5978	-0.1039	-8.1
**b130**	13427.0343	13426.8845	-0.1497	-11.2
**b164**	17213.0963	17212.9301	-0.1662	-9.7
**b182**	18993.1434	18992.9855	-0.1579	-8.3
**y6**	765.4207	765.4236	0.0029	3.8
**y7**	880.4477	880.4459	-0.0017	-2.0
**y11**	1322.7019	1322.6998	0.0021	-1.6
**y14**	1593.8548	1593.8408	-0.0141	-8.8
**y18**	2061.1292	2061.1107	-0.0185	-9.0
**y20**	2245.2504	2245.2596	0.0092	4.1
**y21**	2374.2930	2374.2635	-0.0295	-12.4
**y22**	2445.3301	2445.3249	-0.0051	-2.1
**y23**	2558.4141	2558.4003	-0.0138	-5.4
**y24**	2689.4546	2689.4514	-0.0033	-1.2
**y26**	2946.5922	2946.5802	-0.0119	-4.1
**y29**	3319.7559	3319.7565	0.0006	0.2
**y30**	3420.8036	3420.7723	-0.0314	-9.2
**y31**	3533.8877	3533.8903	0.0026	0.7
**y33**	3719.0041	3718.9926	-0.0115	-3.1
**y35**	3889.1096	3889.0749	-0.0348	-8.9
**y37**	4116.2366	4116.2203	-0.0163	-4.0
**y41**	4543.4797	4543.4546	-0.0251	-5.5
**y44**	4856.6911	4856.6697	-0.0214	-4.4
**y48**	5292.9960	5292.9648	-0.0312	-5.9
**y49**	5394.0437	5394.0297	-0.0140	-2.6

**y50**	5481.0757	5481.0439	-0.0319	-5.8
**y54**	5995.2643	5995.2021	-0.0622	-10.4
**y56**	6225.3546	6225.3254	-0.0292	-4.7
**y57**	6354.3972	6354.3919	-0.0053	-0.8
**y59**	6569.5242	6569.4830	-0.0412	-6.3
**y60**	6697.5827	6697.5305	-0.0522	-7.8
**y61**	6796.6512	6796.6061	-0.0451	-6.6
**y68**	7531.9773	7531.9224	-0.0549	-7.3
**y96**	10334.3242	10334.2450	-0.0792	-7.7
**y97**	10405.3613	10405.2900	-0.0712	-6.8
**y114**	12187.3612	12187.2128	-0.1484	-12.2
**y117**	12499.5409	12499.4693	-0.0717	-5.7
**y125**	13425.9907	13425.9533	-0.0375	-2.8
**y132**	14168.3591	14168.3513	-0.0078	-0.5
**y150**	15948.4062	15948.3854	-0.0208	-1.3
**y181**	19331.2099	19331.1069	-0.1029	-5.3
**y190**	20344.8035	20344.8329	-0.0294	1.4
**y198**	21157.2036	21157.0005	-0.2031	-9.6
**y206**	22027.6406	22027.6861	-0.0455	2.1

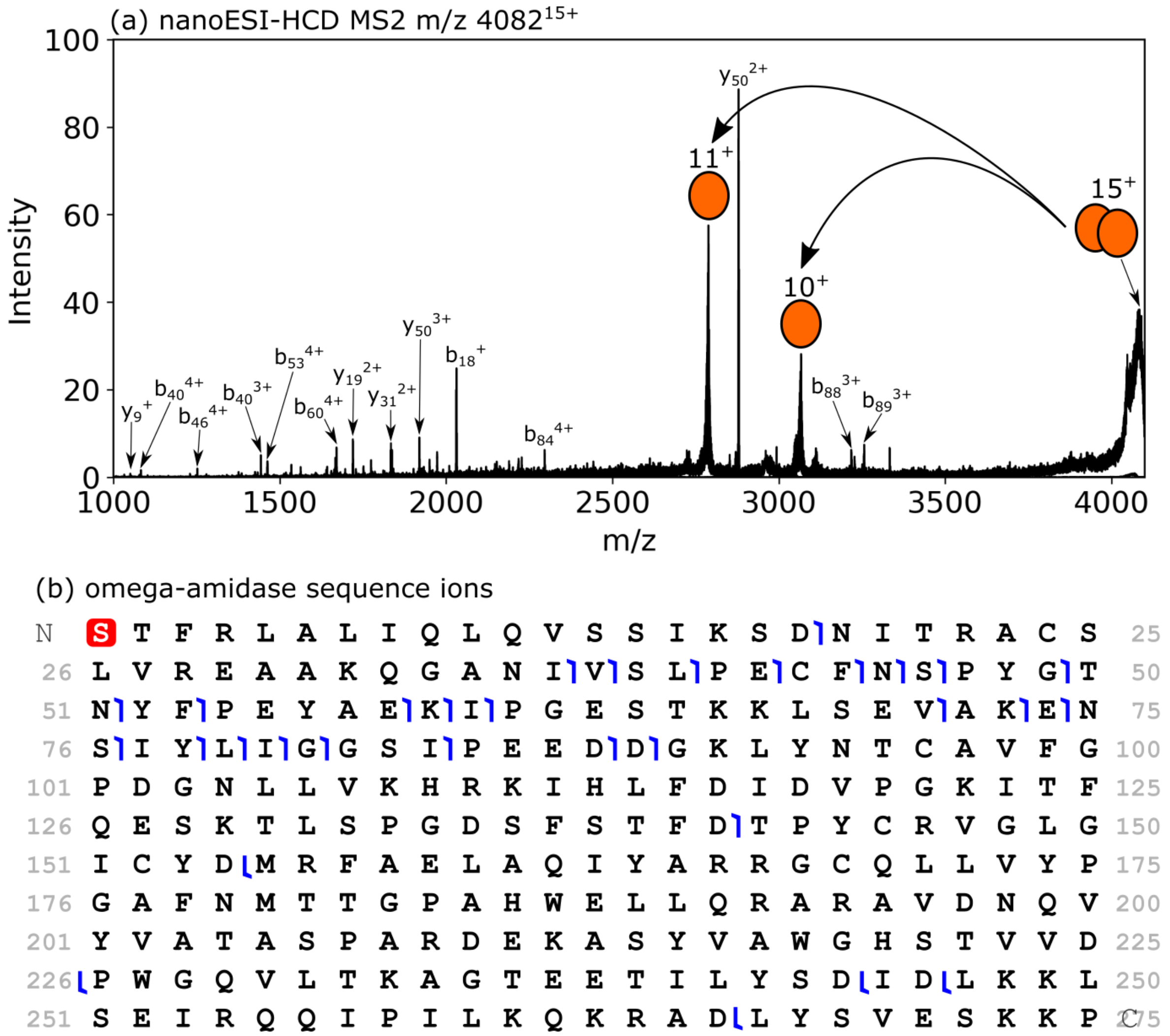

Figure S12: (a) nano-ESI-HCD MS2 spectrum showing subunit signals and sequence ions of omega-amidase. Subunit ions confirm the assembly stoichiometry as homodimeric.. (b) Sequence ions of N-terminally acetylated omega-amidase automatically assigned with a mass tolerance of 20 ppm. P-score = 3.3e-15. 14 of 31 ions are explained by cleavage at D or P residues. Data were acquired on the Orbitrap Eclipse, (NCE = 46 – 55%) using a 200 mM ammonium acetate + 0.5x CMC C8E4 solvent system.

Table S9: sequence ions for omega-amidase

Name	Theoretical Mass (Da)	Observed Mass (Da)	Mass Difference (Da)	Mass Difference (ppm)
**b18**	2029.1208	2029.1123	-0.0085	-4.2
**b37**	4025.1844	4025.1479	-0.0365	-9.1
**b38**	4124.2528	4124.2083	-0.0444	-10.8
**b40**	4324.3689	4324.3262	-0.0427	-9.9
**b42**	4550.4642	4550.4378	-0.0265	-5.8
**b44**	4800.5418	4800.4908	-0.0510	-10.6
**b45**	4914.5847	4914.5425	-0.0423	-8.6
**b46**	5001.6168	5001.5996	-0.0172	-3.4
**b49**	5318.7543	5318.7069	-0.0475	-8.9
**b51**	5533.8449	5533.7869	-0.0580	-10.5
**b53**	5843.9767	5843.9466	-0.0301	-5.1
**b58**	6433.2151	6433.1711	-0.0439	-6.8
**b59**	6561.3100	6561.2365	-0.0735	-11.2
**b60**	6674.3941	6674.3311	-0.0630	-9.4
**b71**	7830.0076	7829.9635	-0.0441	-5.6
**b73**	8029.1397	8029.0828	-0.0568	-7.1
**b74**	8158.1823	8158.1063	-0.0759	-9.3
**b76**	8359.2572	8359.2038	-0.0534	-6.4
**b78**	8635.4046	8635.3360	-0.0686	-7.9
**b79**	8748.4887	8748.3975	-0.0912	-10.4
**b80**	8861.5727	8861.4898	-0.0830	-9.4
**b81**	8918.5942	8918.5110	-0.0831	-9.3
**b84**	9175.7317	9175.6589	-0.0729	-7.9
**b88**	9645.8966	9645.8248	-0.0719	-7.4
**b89**	9760.9236	9760.8289	-0.0947	-9.7
**b141**	15494.8528	15494.6840	-0.1688	-10.9
**y9**	1049.5760	1050.6445	-1.0685	-6.8
**y29**	3436.0293	3435.9857	-0.0436	-12.7
**y31**	3664.1403	3664.0969	-0.0434	-11.8
**y50**	5753.1770	5753.1382	-0.0389	-6.8
**y121**	13657.1237	13656.9793	-0.1443	-10.6

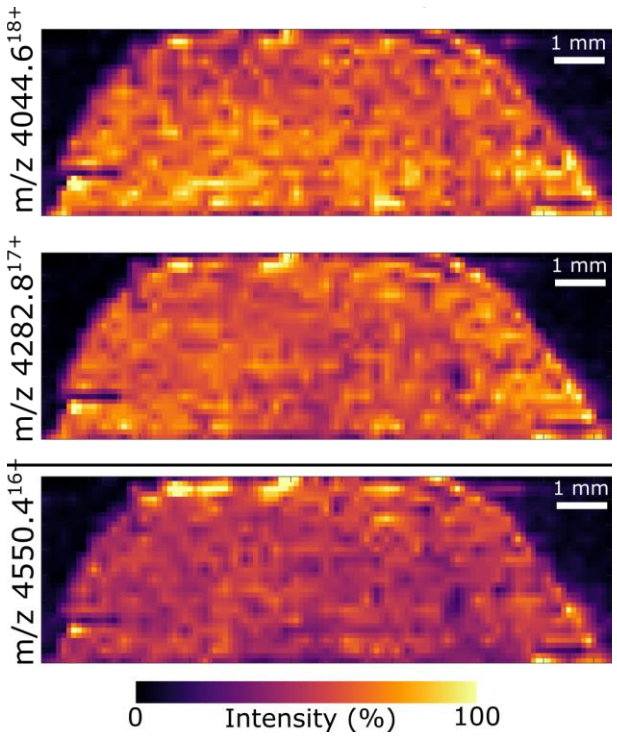

Figure S13: ion images for the 18+, 17+ and 16+ charge states of the MDH1 homodimer showing homogenous distribution.

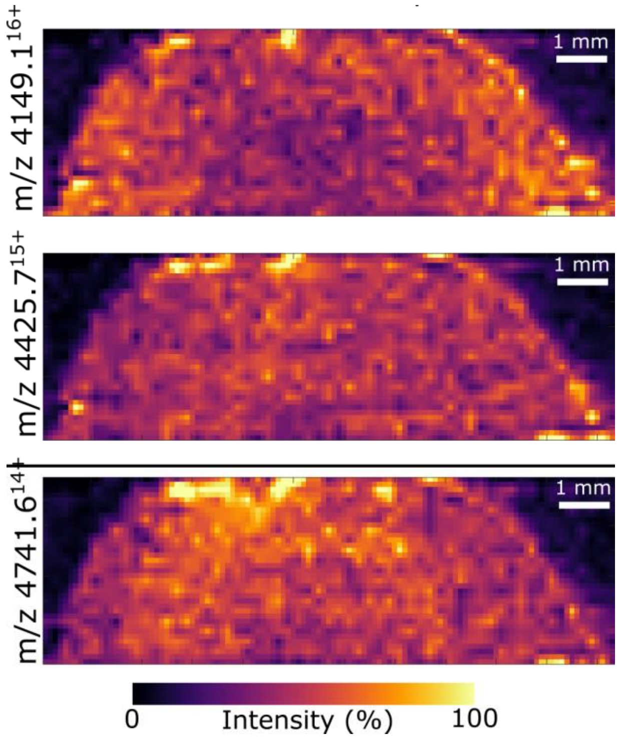

Figure S14: ion images for the 16+, 15+ and 14+ charge states of MDH2 showing homogenous distribution.

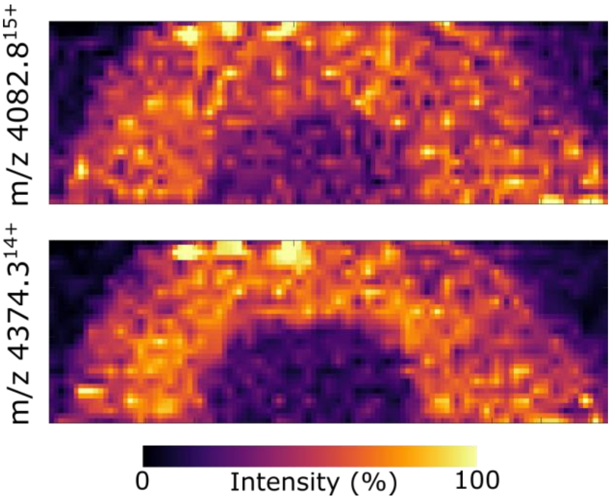

Figure S15: ion images for 15+ and 14+ charge states of omega-amidase homodimer showing distribution throughout cortex tissue.

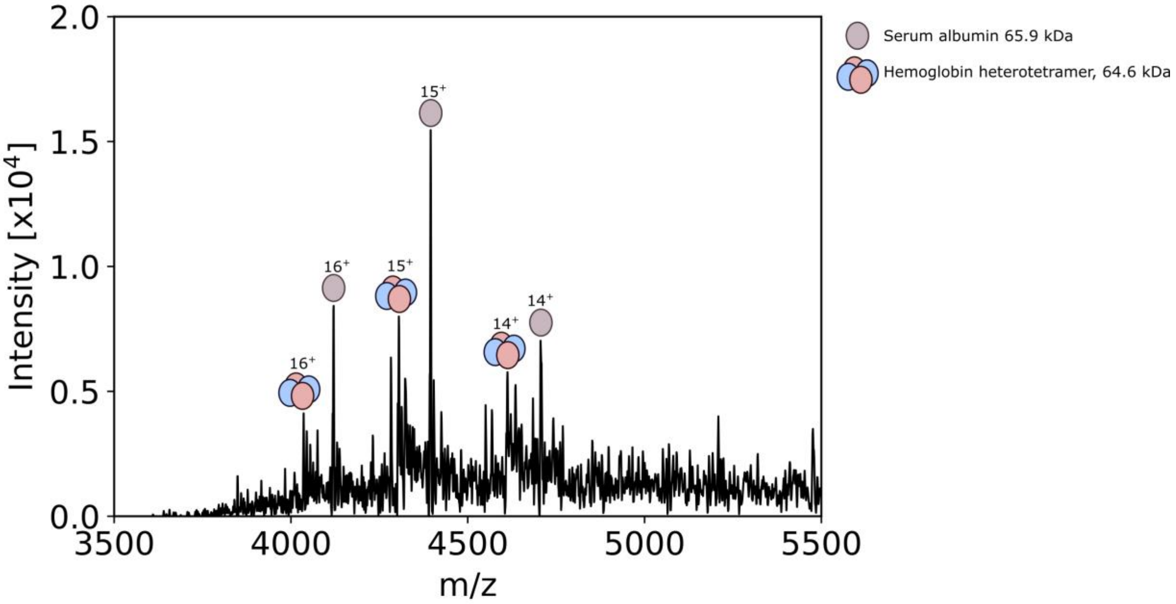

Figure S16: native mass spectrum composed of the average signals from 12 scans in a vascular region by nano-DESI. Signals for serum albumin and intact hemoglobin heterotetramer dominate the spectrum.

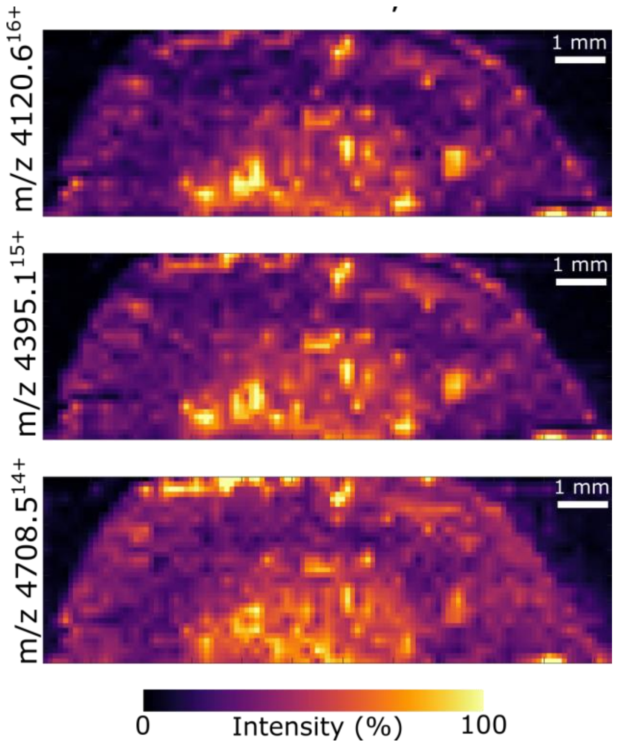

Figure S17: ion images for 16+, 15+ and 14+ charge states of serum albumin showing distribution in vascular regions and the medulla.

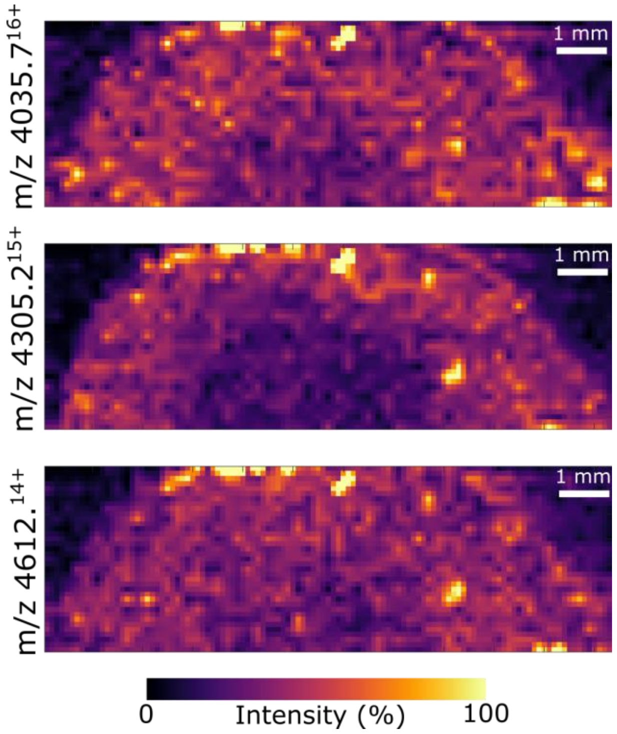

Figure S18: ion images for 16+, 15+ and 14+ charge states of hemoglobin heterotetramer showing distribution in blood vessels.

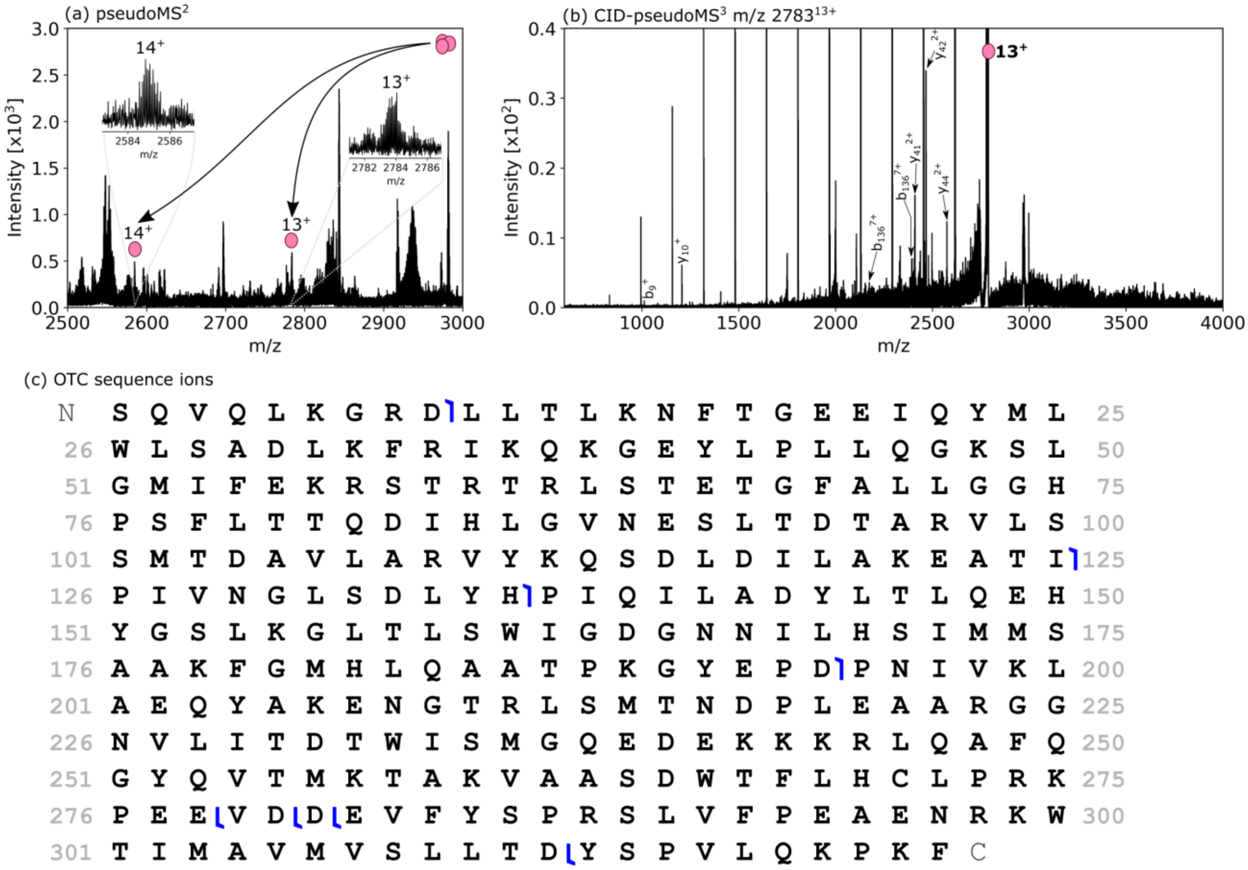

Figure S19: (a) nanoESI-pseudoMS^2 by in-source fragmentation enabled detection of isotopically resolved OTC monomers consistent with the intact assembly being a homotrimer (Data were acquired on the Orbitrap Eclipse at orbitrap resolution = 500,000 at m/z 200). (b) NanoESI-pseudoMS^3 by isolation and CID fragmentation of the 13+ monomer provided main sequence product ions from OTC. P-score = 0.15. 8 of 10 sequence ions are explained by cleavage at D or P residues. The regular intense peaks are attributable to product ions from carbohydrate precursor ions co-isolated with OTC 13+ which give a repeating neutral loss of -162Da. (c) Sequence ions assigned with a mass tolerance of 20 ppm.

Table S10: sequence ions for OTC.

Name	Theoretical Mass (Da)	Observed Mass (Da)	Mass Difference (Da)	Mass Difference (ppm)
**b9**	1011.5464	1011.5478	-0.0014	-5.5
**b125**	13992.4582	13992.4066	-0.0516	-3.7
**b136**	15201.0772	15200.8124	-0.2648	-17.4
**b194**	21537.2621	21537.1460	-0.1161	-5.4
**y10**	1205.6808	1205.6773	-0.0035	-2.9
**y41**	4815.5172	4815.4878	-0.0294	-6.1
**y42**	4930.5441	4930.5140	-0.0301	-6.1
**y44**	5144.6394	5144.6696	0.0302	5.9

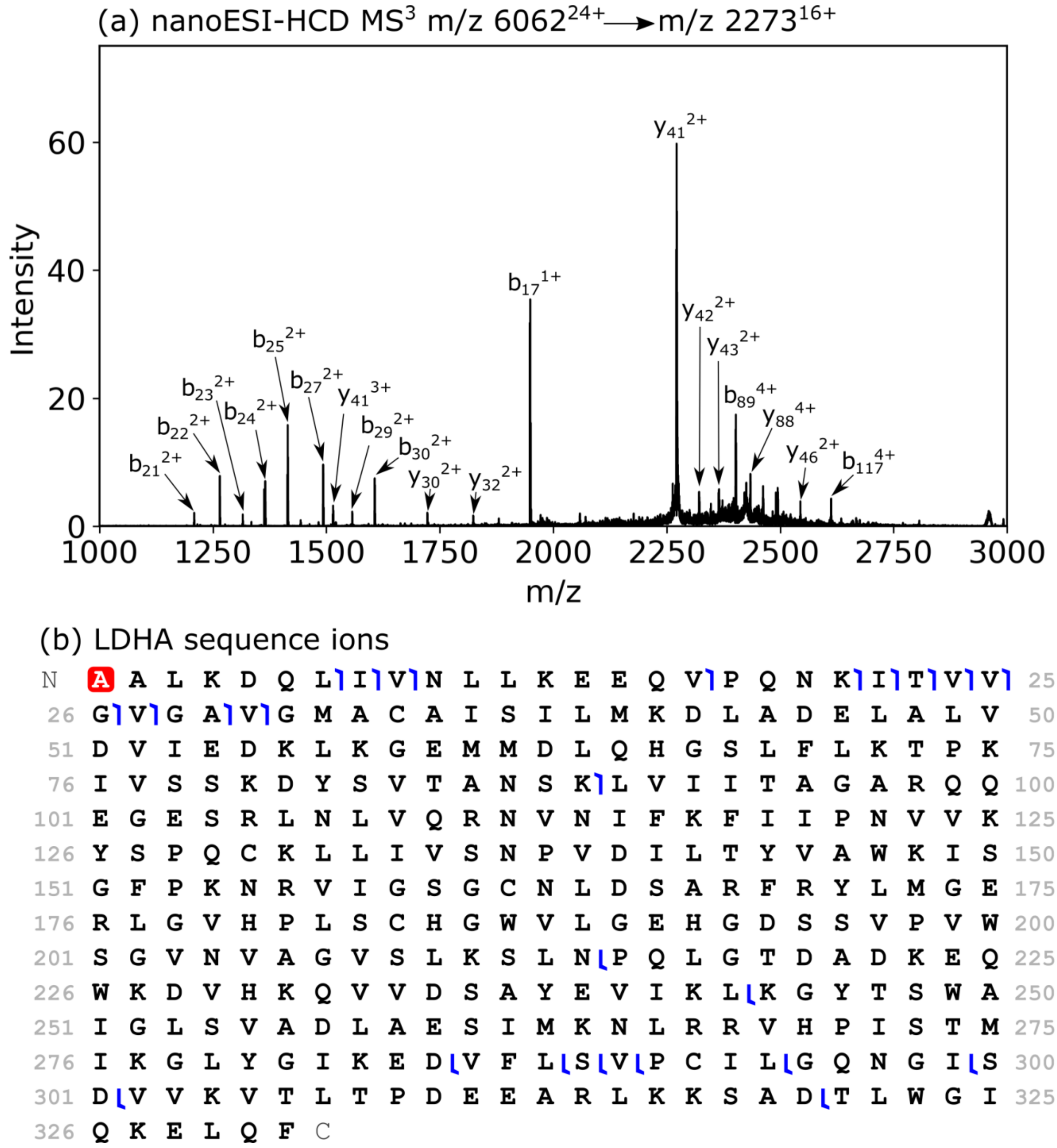

Figure S20: (a) nanoESI-HCD MS3 of LDHA. Tetramers were dissociated to monomers by HCD (NCE = 40%) then the monomer was isolated and dissociated by HCD (NCE = 60%). (b) LDHA sequence ions assigned within a mass tolerance of 20 ppm. P-score = 3.5e-10. 6 of 24 sequence ions were the result of cleavage at the C-terminus of D or N-terminus of P.

Table S11: Sequence ions for LDHA

Name	Theoretical Mass (Da)	Observed Mass (Da)	Mass difference (Da)	Mass Difference (ppm)
**b7**	781.4334	781.4385	-0.0051	-2.8
**b8**	894.5174	894.5222	-0.0048	-2.8
**b17**	1947.1040	1947.112	-0.0080	-3.4
**b9**	993.5859	993.5897	-0.0038	-3.5
**b21**	2414.3532	2414.36	-0.0064	-3.4
**b22**	2527.4374	2527.443	-0.0058	-3.5
**b23**	2628.4850	2628.492	-0.0070	-2.9
**y11**	1361.7343	1361.737	-0.0025	-3.5
**b24**	2727.5534	2727.561	-0.0074	-2.6
**b25**	2826.6218	2826.626	-0.0046	-3.5
**b26**	2883.6432	2883.649	-0.0056	-3.1
**b27**	2982.7118	2982.716	-0.0042	-3.5
**y41**	4539.4048	4539.411	-0.0059	-3.5
**b29**	3110.7702	3110.772	-0.0014	-4.2
**b30**	3209.8388	3209.842	-0.0028	-3.7
**y30**	3441.8872	3441.883	0.0044	-5.5
**y32**	3643.9460	3643.971	-0.0246	2.7
**b17**	1947.1041	1947.105	-0.0006	-3.4
**y37**	4113.1746	4113.189	-0.0146	0.0
**y41**	4539.4046	4539.403	0.0018	-3.6
**y42**	4638.4730	4638.463	0.0096	-5.2
**y43**	4725.5050	4725.524	-0.0194	1.0
**b89**	9601.1289	9601.1	0.0285	-6.0
**y88**	9727.2029	9727.279	-0.0759	4.8
**y46**	5084.7260	5084.689	0.0370	-10.1
**y117**	13047.9071	13047.92	-0.0161	-1.6

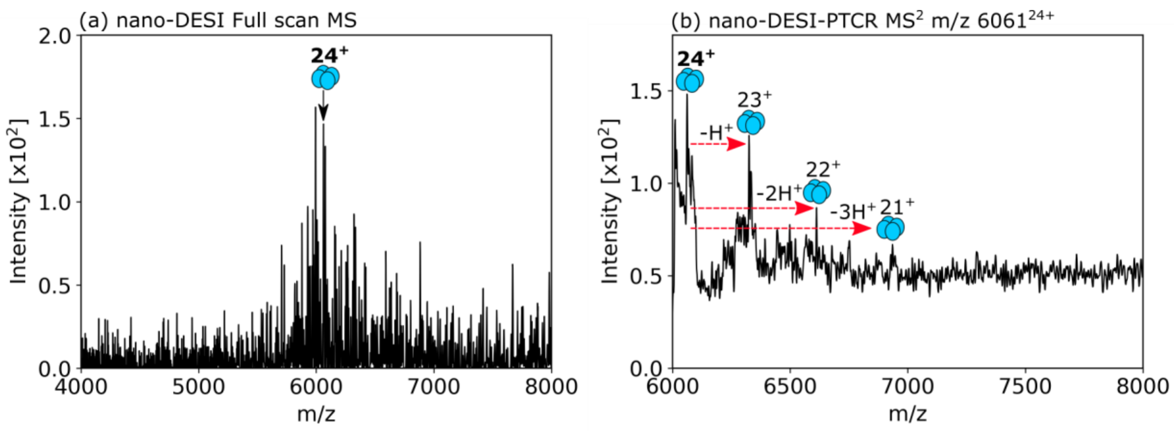

Figure S21: nano-DESI analysis of LDHA in kidney tissue (a) full scan mass spectrum with 24+ charge state detected and (b) PTCR MS2 spectrum of m/z 6061^{24+} enabling confirmation of charge state and intact mass. The data is much noisier than the equivalent data obtained with nanoESI. Data were acquired on the Orbitrap Eclipse at an orbitrap resolution of 7500 at m/z 200.

References

(1) Randall, E. C.; Bunch, J.; Cooper, H. J. Direct analysis of intact proteins from Escherichia coli colonies by liquid extraction surface analysis mass spectrometry *Anal Chem* **2014**, *86*, 10504-10510.
